# Supplementary material for: Closing Kok’s cycle of nature’s water oxidation catalysis
Source: Nat Commun. 2024 Jul 16;15:5982. doi: 10.1038/s41467-024-50210-6 (PMC11252165; doi:10.1038/s41467-024-50210-6)
Supplement: Supplementary file 1 — Supplementary Information [file 41467_2024_50210_MOESM1_ESM.pdf]

Supplementary information

Closing Kok's cycle of nature's water oxidation catalysis

Yu Guo[1,2], Lanlan He[1,2], Yunxuan Ding[1,2], Lars Kloo[3], Dimitrios A. Pantazis[4], Johannes Messinger[5,6], Licheng Sun[1,2,7*]

[1]*Center of Artificial Photosynthesis for Solar Fuels and Department of Chemistry, School of Science, Westlake University, Hangzhou 310024, China.*

[2]*Institute of Natural Sciences, Westlake Institute for Advanced Study, Hangzhou 310024, China.*

[3]*Department of Chemistry, School of Engineering Sciences in Chemistry, Biotechnology and Health, KTH Royal Institute of Technology, SE-10044 Stockholm, Sweden.*

[4]*Max-Planck-Institut für Kohlenforschung, Kaiser-Wilhelm-Platz 1, 45470 Mülheim an der Ruhr, Germany.*

[5]*Department of Plant Physiology, Umeå University, Linnaeus väg 6 (KBC huset), SE-90187, Umeå, Sweden.*

[6]*Molecular Biomimetics, Department of Chemistry – Ångström Laboratory, Uppsala University, SE-75120 Uppsala, Sweden.*

[7]*Division of Solar Energy Conversion and Catalysis at Westlake University, Zhejiang Baima Lake Laboratory Co., Ltd., Hangzhou 310000, Zhejiang, China.*

***Correspondence:**

sunlicheng@westlake.edu.cn (Licheng Sun)

Contents

I. Supplementary Notes

Suppl. Note 1. O5 and Ox as substrates for the present study……………….………..5

Suppl. Note 2. Clarification on the stage simulated by BO-AIMD…………………..7

Suppl. Note 3. About the protonation state of W2……………………………….....7

Suppl. Note 4. Rationality of $Im0^{-O_2}$ as the starting state for water insertion……......9

Suppl. Note 5. Spin state definition………………………………………...………..10

Suppl. Note 6. Structural changes and energetics for the 'pivot/carousel'-like reorganization………………………………………………………………………...11

Suppl. Note 7. More interpretation on the simulation results related to model construction…………………………………………………………………………..13

Suppl. Note 8. Energetics from $Im0^{-O_2}$ to Im2 related to simulation limitation……………………………………………………………………….…16

Suppl. Note 9. The proton release in the S_0 restoration……………………….......20

Suppl. Note 10. Reflections on W2 dissociation from Mn4(III) in the pre-S_0 state……………………………………………………………………………...21

Suppl. Note 11. Effect of dispersion parameters…………………………..……......24

Suppl. Note 12. Relative population of the S_0 isomers related to calculated energetics…..25

Suppl. Note 13. Validity of the closed-cubane structure in the catalytic cycle……...27

Suppl. Note 14. Complementary explanation for the truncated model construction…………………………………………………………………………..30

Suppl. Note 15. General scheme of IRC and comparison with NEB………...……..32

Suppl. Note 16. Comparison with femtosecond X-ray crystallography during O_2 formation……………………………………………………………………..…35

Suppl. Note 17. Roles of specific water molecules and hydrogen-bond interaction…………………………………………………………………………37

Suppl. Note 18. Comparison to previous reports……………………………...…....40

II. Supplementary Tables

Suppl. Table 1. Gibbs free energies of Im0$^{-O_2}$, Im1 and Im2 for the octet/αααβ and doublet/αβαβ spin states…………………………………………………………...42

Suppl. Table 2. Reaction energetics of W1(H_2O) deprotonation to Asp61 for the octet/αααβ and doublet/αβαβ spin states…………………………..……………...43

Suppl. Table 3. Gibbs free energies of pre-S_0(W2-bound), TS(Mn4-W2) and pre-S_0(W2-unbound) for the octet/αααβ and doublet/αβαβ spin states………………….…44

Suppl. Table 4. Gibbs free energies of S_0^B(closed-cubane), TS(Mn1-W3-Mn4) and S_0^A(open-cubane) for the octet/αααβ and doublet/αβαβ spin states…………….………45

Suppl. Table 5. Gibbs free energies of pre-S_0(W2-bound), TS(Mn4-W2) and pre-S_0(W2-unbound) for W2 dissociation, and S_0^B(closed-cubane), TS(Mn1-W3-Mn4) and S_0^A(closed-cubane) for the S_0 isomerism under the doublet/αβαβ spin state by different functionals…..…………………………………………………………...…46

Suppl. Table 6. Gibbs free energies of S_0^B(closed-cubane), TS and S_0^A(closed-cubane) for the S_0 isomerism by using the model including D1-Asp61 truncated from 6DHP, under the doublet/αβαβ spin state by different functionals……………..…………...47

III. Supplementary Figures

Suppl. Fig. 1. The initial BO-AIMD model for water insertion dynamics truncated from 6W1V, after removal of O5 and Ox for O_2 release……………………………..48

Suppl. Fig. 2. Distributions of Mn Mulliken charge populations with time evolution along the BO-AIMD simulation trajectory for the octet/αααβ spin state……..……...48

Suppl. Fig. 3. Distributions of Mn Mulliken charge populations with time evolution along the BO-AIMD simulation trajectory for the doublet/αβαβ spin state………....49

Suppl. Fig. 4. Distributions of Mn Mulliken spin populations with time evolution along the BO-AIMD simulation trajectory for the octet/αααβ spin state…..………...49

Suppl. Fig. 5. Distributions of Mn Mulliken spin populations with time evolution along the BO-AIMD simulation trajectory for the doublet/αβαβ spin state………...50

Suppl. Fig. 6. Distributions of the total energy E_{tot} with time evolution along the BO-AIMD simulation trajectory for the octet/αααβ spin state……………………...50

Suppl. Fig. 7. Distributions of the total energy $E_{\_tot}$ with time evolution along the BO-AIMD simulation trajectory for the doublet/αβαβ spin state..............................51

Suppl. Fig. 8. The truncated DFT model for W1 deprotonation based on the last snapshot from the BO-AIMD simulation..51

Suppl. Fig. 9. The truncated DFT model for W2 dissociation after W1 deprotonation...52

Suppl. Fig. 10. The truncated DFT model for W3 shift after W2 dissociation...........52

Suppl. Fig. 11. Functional dependence of the reaction energetics for (a) water dissociation in the pre-S_0 state and (b) the subsequent structural isomerism in the S_0 state under the doublet/αβαβ spin state using different functionals............................53

Suppl. Fig. 12. The DFT model truncated from 6DHP including Asp61 for μ-O5H shift…………………………………………………..54

Suppl. Fig. 13. Functional dependence of the reaction energetics of the structural isomerism in the S_0 state by using the model including D1-Asp61 truncated from 6DHP under the doublet/αβαβ spin state using different functionals………………..54

Suppl. Fig. 14. The IRC curve for W1 deprotonation to Asp61 for the octet/αααβ spin state 55

Suppl. Fig. 15. The IRC curve for W1 deprotonation to Asp61 for the doublet/αβαβ spin state...56

Suppl. Fig. 16. The IRC curve for W2 dissociation for the octet/αααβ spin state......57

Suppl. Fig. 17. The IRC curve for W2 dissociation for the doublet/αβαβ spin state...58

Suppl. Fig. 18. The IRC curve for the cluster isomerization in the S_0 state for the octet/αααβ spin state.. ………………………………………………………….…....59

Suppl. Fig. 19. The IRC curve for the cluster isomerization in the S_0 state for the doublet/αβαβ spin state…………………………………………………..………...60

IV. The Fortran code used to collect the interatomic distances……..………61

V. Supplementary References…………………………………………………...62

I. Supplementary Notes

Suppl. Note 1. O5 and Ox as substrates for the present study

Until now, no experimental techniques have directly observed the O-O bond formation itself in the OEC, and the peroxide intermediate formed by oxo-oxyl coupling has not yet been confirmed experimentally. Bhowmick et al. did not capture the peroxide intermediate (typical O-O distance 1.4 to 1.5 Å) by XFEL crystallography for the timepoints during the S_3-S_0 transition, and their indication for the peroxide-like species is mostly suggested by the timescale and kinetics for the observed structural changes of the cluster (there is a delay between the onset of O-O bond formation and the decrease of the Ox electron density)[1]. The evidence of oxo-oxyl coupling from Greife et al. is to some degree embodied from the computational results[2]. Therefore, different peroxide intermediates may form different structures after O_2 release and influence the following reset process. It can be seen that up to now uncertainties still remain in this process which are unable to be figured out by experimental techniques at least for now. The highlights the important role of computational studies as an auxiliary tool to investigate the section that is unreachable by experiments. Aware of the existing uncertainties, we have to make careful deliberation and select the most likely scenario as the foundation of our study, according to the majority of available evidence. Specifically here, it is obvious that we have selected the most likely mechanism for O-O bond formation as the base for the following Mn_4CaO_5 resetting process. So far, the preponderance of available evidence from most experimental and theoretical studies, including XFEL

crystallography[1,3], time-resolved spectroscopies[2], isotope labeling water exchange experiments[4-7], DFT modelling[8-12] and large-scale QM/MM simulations[13-17], etc., strongly supports the oxo-oxyl coupling mechanism (at least for O5 and Ox as substrates). Also, both of the two recent breakthroughs by Bhowmick et al.[18] and Greife et al.[2] (as the background of our present study) have recommended it as the most viable or even compelling pathway, respectively, from the perspectives of structural intermediates by XFEL crystallography and time-resolved microsecond Fourier transform infrared spectroscopy (FTIR) combined with computational chemistry, respectively. Actually, as long as the substrates are O5 and Ox, regardless of how they couple (in terms of electronic configuration of Ox and open/closed conformation of the cluster[6,19-21]) or the variable spatial orientation of the peroxide moiety[13,15], the resetting process suggested in this study is generally applicable because the Mn_4CaO_4 cluster with the structural cavity left by release of O_2 (O5-Ox) is set as the starting point. However, our work does not aim to rule out or decrease the significance of other possibilities of O-O bond formation[22-25] that are worth consideration in parallel. The corresponding cluster resetting process in these scenarios would be totally different, since the structural cavity within the cluster left by O_2 release would not be the same, depending on the position of substrates. They should be studied specifically as well if compelling evidence against the current O5-Ox coupling would appear in future. One should be open-minded about this, but for the present study, we must follow the currently most convincing mechanism of O_2 formation for exploring the subsequent possible routes toward the S_0 state. Thus the

structural basis of the present study focusing on the S_4-S_0 transition makes sense, even if direct experimental evidence capturing the bonded O-O moiety (peroxide intermediate) still lacks. In this putative identification, theoretical studies have particular importance or even play a fundamental role because the crucial parts of the transition remain inaccessible to experimental studies. This is reflected in the present study for the post stage of O_2 formation.

Suppl. Note 2. Clarification on the stage simulated by BO-AIMD

The 'S_3-S_0' transition can be divided into '$S_3 \rightarrow S_4$' and '$S_4 \rightarrow S_0$' transitions. According to Greife et al.[2], '$S_3 \rightarrow S_0$' takes place on a millisecond timescale (ca. 2.5 ms), in which '$S_3 \rightarrow S_4$' for formation of a Mn(IV)-$O^{\bullet}$ radical through single-electron multi-proton transfer is the rate-determining step, and the subsequent '$S_4 \rightarrow S_0$' for O_2 formation and release is ultrafast. The current picosecond BO-AIMD simulations cover only part of the '$S_4 \rightarrow S_0$' transition after O_2 release (from $Im0^{-O_2}$ to Im2), which is proven facile from the rapid structural evolution and the downhill energetic changes as shown in our data (Suppl. Table 1). This means the chemistry from $Im0^{-O_2}$ to Im2 (rather than 'S_3 to S_4 to S_0') occurs on a picosecond timescale, which can be captured in the MD trajectories.

Suppl. Note 3. About the protonation state of W2

For the protonation state of W2, in this study, we follow the hydroxide form (OH^-) that was suggested to be more consistent with the experimental magnetic and electron paramagnetic resonance (EPR) spectroscopic data[26,27] and the pKa predictions by electrostatic energy computations[28]. Although the W2=H_2O scheme cannot be ruled

out[11,29-32], there have been a majority of research work and review papers based on the W2=OH^- scheme[5,8-10,14,16,17,33-50]. Even for Greife et al.'s theoretical section[2], the core proposal of single-electron—multi-proton transfer event relies heavily on the hydroxide (OH^-) nature of W2 for accepting the proton abstracted from O6. Different protonation patterns can lead to discernible spin projections and ^{55}Mn hyperfine coupling parameters, as shown in the computational results with respect to the lower S-states where ENDOR EPR experiments have been conducted. No experiments are available for the higher S-states and certainly not for intermediates that cannot be observed spectroscopically past the S_3 state. Here we list two specific examples supporting W2=OH^-. Ames et al.'s BS-DFT calculations[26] suggest that one of the two water molecules (assigned to W2) that are proposed to coordinate to the outer Mn4 of the cluster is deprotonated in the S_2 state, as this leads to optimal experimental agreement, reproducing the correct ground state spin multiplicity (S=1/2), spin expectation values, and EXAFS-derived metal-metal distances; Krewald et al.[27] have made comprehensive evaluations on considerable models with different oxidation and protonation states with respect to their geometric, energetic, electronic, and spectroscopic properties compared to available experimental EXAFS, XFEL-XRD, EPR, ENDOR and Mn K pre-edge XANES data. While the high-valent scheme is conclusively favored, W2=OH^- (called 'three-proton model' therein) remains the preferred description whereas W2=H_2O ('four-proton model') cannot fit both EPR signals of the S_2 state. Consequently, it is reasonable and valid to assume the model with W2=OH^- for the further modelling on the water insertion process. However, the

issue of the protonation state of W2=H_2O/OH^- is not settled and it is noted that other studies that attempted to reproduce other types of spectroscopies (Fourier transform infrared (FTIR)[51,52] and X-ray absorption spectroscopy (XAS)[53] respectively) favor a doubly protonated W2(H_2O) in certain S states, and there are also other studies adopting W2=H_2O in certain S states[11,23,29,30,54-58].

Suppl. Note 4. Rationality of Im0$^{-O_2}$ as the starting state for water insertion

For the widely accepted open-cubane oxo-oxyl coupling mechanism, three steps are involved (oxo-oxyl→peroxide→superoxide→molecular O_2) in which only one bond is broken in each step. This has been extensively shown in the theoretical studies[9-11,14,21,59]. Five-coordinate Mn(III) can stably exist in the OEC (in the S_0, S_1 and S_2 states), as well as many other Mn model compounds[60-62]. Therefore it is also reasonable to include three 5-coordinate Mn(III) in Im0^{-O2}, pending water insertion. The only point that was under debate is whether O_2 release and water insertion are stepwise or concerted. Siegbahn noticed this issue,[8] where he showed "A large effort was spent trying to find this type of concerted pathway, but without success. There was never any sign of a gain in energy by letting the water enter early". Thereafter he proposed the stepwise model that water insertion occurs after O_2 has been released, and obtained satisfactory energetics for the S_4-S_0 transition which can fit very well to available experimental observations. Later, Shoji et al. proposed a concerted mechanism of water insertion and O_2 release based on QM/MM calculations[63], whereas this hypothesis ignored dynamic effects. Specially for this point, Capone et al. explicitly investigated the sequence of O_2 release versus water insertion by MEP

calculations using QM/MM MD simulations[47]. The energy barrier for the concerted mechanism was found to be ca. 30 kcal mol^{-1}, which is much higher than the two-step mechanism (ca. 14 kcal mol^{-1} and then ca. 4 kcal mol^{-1}) and would be obviously impossible to take place within the ca. 2.5 ms timescale of the S_3-S_0 transition.[2] Consequently, the two-step mechanism is much more favored and acts as the theoretical basis of our starting model $Im0^{-O2}$. Bhowmick et al.'s observation[1] that "there is no missing oxygen density besides Ox (O5 weakened at 1200 μs)" supports that O_2 release and refilling of the empty site are highly coordinated. Here 'highly coordinated' does not conflict with the two-step mechanism. The immediate water binding after O_2 release would make it impossible to capture the $Im0^{-O2}$ intermediate with a sufficiently large accumulation by the XFEL experiment. Furthermore, Bhowmick et al. used several hundreds of microseconds as the time interval for snapshots, which is much longer time step than the picosecond timescale of the structure evolution and thus cannot capture the $Im0^{-O2}$ intermediate. Although the XFEL experiment cannot distinguish between the concerted or stepwise mechanism, the authors seem also supportive for the stepwise mechanism seen from their statement "Once O_2 is released, refilling of the cluster with a new substrate water seems to occur immediately". Furthermore, the two-step mechanism is also reflected in Greife et al.[2], and our starting model $Im0^{-O2}$ coincides with the structure.

Suppl. Note 5. Spin state definition

Because the O-O bond formation and triplet O_2 release in the open-cubane structure of the S_4 state are confined to the sextet/αααββ [Mn1(IV), Mn2(IV), Mn3(IV),

Mn4(IV), Ox•] and doublet/βαβαα spin states under the spin-alignment rule[9,10,15], the corresponding spin states for the O_2-released model should be octet/αααβ [Mn1(III), Mn2(IV), Mn3(III), Mn4(III)] and doublet/αβαβ, respectively, the former of which will undergo spin crossing to the ground spin state (doublet/αβαβ) of the S_0 state[64-71]. The present study follows the 'high oxidation paradigm' of the Mn cluster that has been widely accepted, in contrast to the 'low oxidation paradigm'[27,72].

Suppl. Note 6. Structural changes and energetics for the 'pivot/carousel'-like reorganization

The W1 displacement to become the new W2 while W7 becomes the new W1 are considered as barrierless (or almost) processes since they were observed in our MD simulations to take place spontaneously and complete within only 1 to 2 picoseconds in the transition region from Im1 to Im2; furthermore, they are actually synchronously coupled and show the 'pivot/carousel'-like ligand reorganization around the dangler Mn4, together with W2 rotation after its protonation. The 'pivot/carousel' mechanism was proposed by Retegan et al.[38], Askerka et al.[73-75], and Capone et al.[76] for the S_2-S_3 transition, and the barrier for water binding to Mn4(IV) (from the O4 channel) was reported to be 4.5 (Retegan et al.) and 8 kcal mol^{-1} (Capone et al.) (no barrier data from Askerka et al.), which are sufficiently low to occur but still kinetically slower than the process focused on here. We speculate the more facile ligand reorganization suggested here could be attributed to two factors that are absent in the 'pivot/carousel' mechanism for S_2-S_3: valence (III) of Mn4 and the W3-W2 interaction. The bond strength of ligands (H_2O) on Mn4(III) should be less than that of OH^- on Mn4(IV), so

that ligand displacement around Mn4(III) can take place at lower energy cost. Besides, W2 is connected by the strong HB interaction to W3 which is bonded to Mn1, making W2 rotate around Mn4 to some degree toward the cavity in Im1. W1 is also affected and moves closer to the para-position of O4 for a more stabilized coordination geometry and thus create enough room to accommodate W7. W2 protonation from W3 weakens the 'structural *trans* effect'[77-80] of Mn4 and significantly facilitates W7 binding. These effects are supposed to account for the smooth and barrierless translocations of water ligands on Mn4(III) observed in the MD simulations. Note that because Mn4 moves outwards during this process, the positions of the new W2 and W1 in Im2 somewhat deviate from those of the original ones in $Im0^{-O_2}$ (but will return to normal in S_0^A). In this process, the H-bond interaction between W1 and D61 is actually not broken because the cluster is expanded by the elongated Mn1-Mn4 distance (or enlarged ∠Mn3-O4-Mn4) and the W1-D61 interaction is maintained (although W1 rotates to some degree around Mn4) and this is another clear difference from the 'pivot/carousel' mechanism suggested for S_2-S_3. W1, to become the new W2 (as it is *trans* to O4 in Im2), is H-bonded to a carboxyl oxygen of Asp61 and an amide oxygen of Asp170 throughout the process. In our proposal, Asp61 will extract one proton from the new W2 and release it to the lumen to generate the pre-S_0 state. It is expected that after proton donation from the new W2, the location of Asp61 would be quite flexible and the side chain would largely rotate, as seen in the classical MD simulation by Rivalta et al.[81] and *ab initio* QM/MM MD simulation by Narzi et al.[49], which would then break the H-bond between them. W7 is H-bonded to O4, W8, the

guanidine group of Arg357, the amide oxygen of Asp170 and the alcoholic hydroxyl group of the side chain of Asp170 in $Im0^{-O_2}$ and Im1; after binding to Mn4 as the new W1, its H-bonds to O4 and Arg357 are broken while others remain. This indicates that the loss of stabilization energy from the H-bond interactions can be fully compensated by W7-Mn4 binding.

Suppl. Note 7. More interpretation on the simulation results related to model construction

It is not unlikely that different models and methodologies might lead to different results, typically seen in the applications of QM and QM/MM methods. While it is a matter of choice to use either QM or QM/MM to model the present system, they have both advantages and limitations.[82,83] QM methods provide a rigorous treatment of electronic structure for specific regions of interest within large systems and provide insights into the detailed mechanisms and energetics of chemical processes; QM calculations can be computationally demanding and compromises the model size which may not fully capture the effects of the surrounding environment, such as solvent or protein interactions. QM/MM methods combine the accuracy of QM [23,84]calculations with the efficiency of MM calculations for the surrounding environment, and can therefore capture the effects of the environment more realistically; QM/MM involve approximations and simplifications in the treatment of the QM and MM regions, QM/MM boundary and their interactions, which can introduce some level of artifacts; QM/MM calculations can still be computationally demanding, particularly for large systems (in part due to the difficult self-consistency and convergence between the QM and MM regions) and long simulation time and

consequently normally a small QM region is defined. While there have been already a number of excellent publications on the OEC employing QM/MM models[15,22,49,50,55,63,85,86], for the present study, we have chosen to use *ab initio* (pure QM description) MD on a large model (369 atoms) based on the following considerations. The chemical event in question is focused on the structural evolution of the central Mn cluster under the interaction of water molecules in close proximity involving water binding to Mn/Ca and proton transfer between Mn ligands, and therefore an accurate and intact QM region with as much of the environment as possible around the Mn cluster should be more important and capable to capture the central events in process studied. To the best of our knowledge, the QM size is by far the largest one used among all molecular dynamic studies on the OEC, and the special GPU acceleration customed by the software employed enables a long simulation time even for such a large QM model. Nevertheless, adding the outer MM region based on this large QM core could be in principle an additional factor worth to try, but the performance efficiency and accuracy needs to be investigated. We note there are already a series of excellent QM/MM MD publications in this field[16,47,49,50] but in our work we define a larger QM size with longer simulation time. Regarding the spontaneous movements of W3 binding to Mn1 and W7 binding to Mn4, they are notably observed in our simulations, but they make chemical sense and the simulations have taken into account all the necessary H-bond interactions of the key groups. W3 is H-bonded to W5 and HOH511 which are further H-bonded to W6 which in turn is further H-bonded to Asn181; W7 is H-bonded to O4, W8, the

guanidine group of Arg357, the amide oxygen of Asp170 and the alcoholic hydroxyl unit of the side chain of Asp170; W8 is H-bonded to HOH574, the amide oxygens of Asp170 and Asn87. Since all these H-bonded connected groups are explicitly included in the model by the QM description, their influences on the movement of W3, W7, and W8 should have been properly embodied in the MD simulations. Thus, it can be noted that even for the most distant water molecules such as W6 and W8, the H-bond interactions with other surrounding water molecules or protein residues are also explicitly included. The more distant (approximately >8 Å away from the cluster) water molecules and protein residues that are not included in the model do not have direct H-bond interactions to any of W3, W7 or W8; they may produce very weak electrostatic and van der Waals interactions that we assume as insignificant and therefore will have little impact on the movement of W3, W7, and W8. The propensity of W3 entering the cavity of the cluster is driven by the significantly stabilized structure of the cluster from three unsaturated 5-coordinated Mn(III) (Mn1, Mn3 and Mn4) to generate saturated 6-coordinations. The reason why W3 binding to Mn1 first instead of Mn4 is related to their discrepant charge distributions and the steric effect of the slightly rotated W2 on Mn4, and the H-bond interaction between W2 and W3. W5, which was H-bonded to W3, is pulled by the W3 motion and then occupies the original position of W3 on Ca. W5 is influenced in a similar way and is also reflected in the literatures on the S_2-S_3 transition[87,88]. The subsequent W7 binding to Mn4 is a straightforward outcome of the 'pivot/carousel'-like ligand reorganization around the dangler Mn4, because of the vacant coordination sphere is created toward

the free water W7 along with an expansion of the ∠Mn3-O4-Mn4 angel by the W3 entrance, W1 translocation to *trans*-O4 and W2 protonation further promoting W7 binding to Mn4. W8, which was H-bonded to W7, is pulled by the W7 motion in a way similar to W5. Thus, the observed phenomena especially related to the movement of water molecules around Ca and Mn4 are chemically well grounded regarding all possible interactions involved.

Suppl. Note 8. Energetics from Im0$^{-O_2}$ to Im2 related to simulation limitation

Since the architecture and Mn/Ca coordination sphere of the cluster remain unchanged after ca. 15 ps, meaning substantially completed water insertion dynamics, a simulation time around 30 ps should be adequate for structural evolution of the cluster. The reported free energies differences from Im0$^{-O_2}$ to Im2 (ca. -30 kcal mol^{-1}) are based on the fully optimized (with backbone constraints) structures on the snapshots extracted from the three phases for Im0$^{-O_2}$ (first snapshot), Im1 (last snapshot) and Im2 (last snapshot). The first snapshot for Im0$^{-O_2}$ is for sake of better reflecting the shape of the Mn cluster in the initial stage immediately after the release of O_2. The reasons why single point energies were not adopted is as follows. As shown in Suppl. Figs. 6-7, the energies extracted from the large model used in the BO-AIMD simulations include significant dynamics in weak but plentiful secondary coordination modes involving the protein backbones and explicit water molecules. This causes quite large variation in total energy for the system, and thus statistics in this aspect may be less informative to reliably represent the energy change, even if snapshot averages are used. In any case, it is expected that extending the

simulation time beyond 30 ps, for instance 100 ps or even longer, is likely to give one of the three outcomes: 1) a nice convergence and a flat $E_{\_tot}$, but this seems unlikely because of the many small changes in secondary coordination structures probably rendering a very slow convergence; 2) a continued slow (i.e. rather flat but still slanting) downhill evolution, for the reasons given in 1); 3) the emergence of a long-period oscillation in $E_{\_tot}$ never really settling to a steady $E_{\_tot}$; this seems the most likely situation for large model systems, just like the case here. For these reasons, besides the extensive computational cost, it is not obvious that very long simulations would solve the problem. Furthermore, the MD simulations capture the inherent thermal fluctuations of the system and the energy output corresponds to instantaneous values at different time points with severe randomness. However, in experimental measurements, energies are typically obtained by averaging over multiple measurements or ensemble sampling, which may be better related to local minima on a potential energy surface. Thus in our opinion, while comparing instantaneous MD energies with experimentally averaged energies may not provide a direct correspondence, more reliable energetics should be extracted from the optimized models of snapshots. Geometry optimizations on the obtained snapshots can eliminate unreasonable or exaggerated energy rise/fall brought by some random, excessive and redundant local structural fluctuations which are common for complex systems during MD simulations, and is probably the most sensible strategy to use. Because geometry optimizations result in local minima on the potential energy surface, using neighboring snapshots with similar structures seems unnecessary. Above all, the

purpose for calculating the energetic change from Im0$^{-O_2}$ to Im1 to Im2 is to show the thermodynamic feasibility for the structural evolution of the cluster, and it is inadequate to build a direct corresponding relationship for the energetics to experimental measurements based on the present model, for which other parts and events during the $S_4 \rightarrow S_0$ transition beyond the local structural evolution surrounding the cluster are not explicitly covered, so there seems no point to pursue an accurate energy difference. This is discussed below in detail.

The comparison of the free energy loss from Im0$^{-O_2}$ to Im2 with the energetics of the donor side reactions (from H_2O to P_{680}) of PSII has motivated us to contemplate on the capabilities and limitations of our theoretical simulations when quantitative estimation compared to experimental measurements are discussed. Dau and coworkers have made significant contributions regarding the energetics and kinetics of the S-state transitions[89,90], and Messinger and coworkers have estimated the driving force for $S_4 \rightarrow S_0$[91], based on which the energetics (in ΔG) for the donor side is approximately in the range of 10 to 20 kcal mol^{-1}, thus our calculated free energy loss from Im0$^{-O_2}$ to Im2 (ca. 30 kcal mol^{-1}) seems overestimated. However, considering the model limitation it may be not appropriate to draw a direct corresponding relationship for the obtained free energy loss from Im0$^{-O_2}$ to Im2 with the energetics of the donor side reactions of PSII. While the energy loss mainly accounts for the thermodynamic feasibility of the facile conversion, the causes of the difference should be valued including but possibly not limited to the following aspects. 1) The thermodynamic effect of the release of O_2, especially for the translational entropy

amounting to as much as -12 kcal mol^{-1} [21,59], is not included in the $Im0^{-O_2}$ model. Since O_2 release represents a process of entropy increase (decreasing the degree of randomness), the actual $Im0^{-O_2}$ state should be energetically lower, which decreases the actual free energy difference with Im2. 2) The hydration entropy effects of Ca and Mn4 are not well expressed in the computed energetics. These are roughly estimated to 2 to 3 kcal mol^{-1} and 3 to 4 kcal mol^{-1}, respectively, referring to hydration by a single water molecule at room temperature using experimental data on hydration entropy taken from Marcus[92]. Since hydration causes an entropy decrease (increasing the degree of randomness), the actual Im2 state should be energetically higher, which again decreases the actual free energy difference with the $Im0^{-O_2}$ state. 3) The extensive rearrangement of the hydrogen-bonding network (HBN) in the protein matrix related to the last proton release to the lumen was not (and is incapable to be) taken into account. According to Bhowmick et al.[1], the HBN rearrangement may be the source of the extended timescale of the S_0 restoration observed by femtosecond X-ray crystallography. Greife et al.[2] suggest a significant entropic slowdown for the S_3-S_4 transition associated with the HBN rearrangement caused by the proton release during the S_3-S_4 transition. While it is clear that the HBN rearrangement can slow down the overall kinetics by destabilizing the rate-limiting TS (by 6.5 kcal mol^{-1} entropic contribution), it may affect the thermodynamics of an intermediate or final state in a similar way. Since proton release is not completed in Im2, the above effect should be deducted when compared with the energetics of the donor-side reactions. Note that even the QM/MM model including the outer protein environment has the

same limitation in characterizing the entropic effect in HBN rearrangement coupled to protein dynamics[2]. 4) The computed energetics do not fully represent the complete donor-side reactions, but only the intrinsic/local energetics of the conformational evolution occurring at/around the inorganic cluster. Limited by the model size and current computation capability, it is difficult to make a computational prediction on numerically how large an effect from other donor-side reactions would exert on the energetic difference between $Im0^{-O_2}$ and Im2; however, the above analyses show plausible reasons for the sources of the discrepancy for quantitative comparison with the energetics of the donor-side reactions, which, if possible to take into account somehow, would significantly narrow the gap. Consequently, while the above factors leading to the computational overestimate within the model imitations are beyond the scope of our simulations, caution should be given when comparing with experimental data. Finally, we emphasize the validity of the structural evolution of the Mn cluster from $Im0^{-O_2}$ to Im2 and that the resulting central conclusion remains unaffected.

Suppl. Note 9. The proton release in the S_0 restoration

For the following reasons, we consider that W1(H_2O) should be the deprotonation site which releases H^+ to the lumen *via* Asp61: 1) W1 becomes the new W2 in Im2 since it is translocated to the *trans* position of O4 by the ‘pivot/carousel’-like ligand reorganization; 2) W2 is generally considered as a hydroxide for the metastable forms of the S states[8,17,26,27,39,41,47]; 3) W1 is in strong hydrogen-bonding interaction with Asp61 which is generally recognized as the gate for proton release[1,2,17,29,93-95]. Further proton release from Asp61 to other groups and then to the lumen along the proton

channel is considered feasible and was not investigated because 1) the function of Asp61 releasing H^+ has been widely acknowledged; 2) the rearrangement of the HB network in the proton channel should have little effect on the structural evolution of the Mn cluster; 3) the residues and water molecules in the proton channel are not sufficiently included in the model since it is not in the focus of this study. In addition, this can represent one of the reasons for why the computed energetics going from $Im0^{-O_2}$ to Im2 may not represent the full donor-side reactions in PSII and thus is inherently overestimated.

Suppl. Note 10. Reflections on W2 dissociation from Mn4(III) in the pre-S_0 state

After formation of the pre-S_0(W2-bound) state upon H^+ release in Im2 (whose rationality as an intermediate has been discussed above), assigning W2 dissociation as the next step is driven by the progression to the open-cubane S_0 state that has been experimentally identified. Thus it is assumed as a necessary step before the structural isomerization of W3(OH^-) ligand transfer. Another point regarding the limits of the calculations concerns the precision of the reaction energetics of W2 unbinding. Application of DFT methodology on a chemical reaction in transition metal chemistry that has been well-addressed in many papers[96-103]. According to previous studies[83,104], QM model with such a size is adequate to yield accurate reaction energetics and spectroscopic properties for the OEC system. Due to the approximations in the accuracy of DFT and the model size limitations using a cluster model,[83,96,98] errors of the obtained energetics are normally estimated to be within a few kcal mol^{-1}, and could be varying among different choices of computational details. It is acknowledged

in many studies/benchmarks that DFT computations can give quite reliable results with errors normally within the range of approximately 1 to 3 kcal mol^{-1} as compared to more advanced (but much more expensive) methods. As shown in Fig. 4, a low-barrier route for W2(H_2O) dissociation from Mn4(III) has been located and the energic barriers for W2 decoupling are calculated to be 4 to 5 kcal mol^{-1} and endothermic for 2 to 3 kcal mol^{-1} by a truncated DFT model using MEP-IRC calculations including 200+ atoms. We have two different spin state models extracted from two different simulation trajectories which obtained very similar energetics for W2 decoupling from, thus it is expected that multiple sampling would not change the situation and the error in the estimated energy would not be significant to affect the conclusion. Practically, more meaningful assessment on the results can be performed by sensitivity tests using different functionals, with the basic motivation that different functionals represent different approximations that could affect the quantitative results obtained. This has been shown in Suppl. Tables 5-6 and Suppl. Fig. 11 together with the sensitivity tests for the isomerism. For W2 decoupling you concern, five more GD3BJ-parameterized DFT functionals yield 3.4 to 4.9 kcal mol^{-1} barriers and endothermic for 2.5 to 4.0 kcal mol^{-1}, which means merely 1 to 2 kcal mol^{-1} deviation. For the isomerism, even changing the source of the model by employing 6DHP of the original S_0 state yields very close energetics (deviation within 1 kcal mol^{-1}), which indicates very small range which would not affect the qualitative conclusion and also reliability of the model used. Besides, the obtained barrier height for W2 detaching from Mn4(III) is quite similar to a recent report on a biomimetic polyoxometalate

water oxidation catalyst[105], indicating rationality for the obtained energetics. Since there are very few referable benchmarks associated to experimental data for special cases, the significance of these values may be more qualitative than quantitative, but they do not undermine the validity of the conclusions which reside within the error bars. Despite unavoidable model artifacts and errors, we consider the dissociation of W2(H_2O) at this state as a chemically sensible process. The transfer of W2 to the bulk is, in terms of behavior, very similar to the last step of the proposed mechanism of water exchange for the OEC. In the model calculation, the Mn4-W2 distance is changed from 2.18 Å (W2 bound) to 3.99 Å (W2 unbound). A distance of ca. 4 Å is generally considered to be in the water exchange region, and in any case, to move the water molecule even further out costs very little energy. Due to the size limitation of the model, only a finite value ca. 4 Å of the Mn4-W2 distance can be obtained but this distance adequately illustrates its tendency to transit to the bulk. The situation and explanation are in analogy to substrate water exchange in the S_1, S_2, and S_3 states proposed by Siegbahn.[33] For the S_0 state, there are still no computational studies on the water exchange mechanism, nor on the exit of W2 in any S_0 structure in literatures. However, in essence they all share the common point that Mn4(III) in a closed-cubane structure serves as the station for water dissociation. Furthermore, in a scheme from the Messinger group based on the experimental data (in their Scheme 3), W2 unbinding from Mn4 is involved in a closed-cubane structure in their hypothetical water exchange mechanism for the S_0 state.[43] That means that W2 departure from Mn4(III) from the cluster represents a realistic model under such circumstances.

Suppl. Note 11. Effect of dispersion parameters

The reason to use the hybrid B3LYP* as the primary choice is that in Siegbahn's benchmark tests B3LYP* shows good agreement with experiments for the OEC. The performance by nonhybrid functionals is not satisfactory because of accumulation of error upon consecutive oxidation steps of Mn(III) to Mn(IV)[106]. However, since Mn oxidation is not involved in any process in this study, more functionals can be considered for comparison where necessary, as shown in *Suppl. Tables* 5-6 and *Suppl. Figs.* 11 and 13. Dispersion effects, also known as van der Waals interactions, play a crucial role in transition metal complexes[107], and it is highly recommended to incorporate Grimme's empirical formula for accurate modeling of these interactions[108-110]. An established approach in the absence of DFT-D3(BJ) dispersion parameters for B3LYP* is to adopt the dispersion parameters developed for standard B3LYP. Despite B3LYP* having a slightly reduced Hartree-Fock exact exchange (around 5% less compared to standard B3LYP), it is expected to introduce only marginal artifacts in the results. Additionally, B3LYP* has been commonly combined with Grimme's dispersion correction in the literature. An example of the consistent usage of B3LYP*-D2 in studies on the oxygen-evolving complex (OEC) systems is seen in the work conducted by Siegbahn[8,9,21,33,111-113]. It is important to note that there are no D2 dispersion parameters specifically developed for B3LYP*, yet Siegbahn has consistently employed this combination in their research. Additionally, a recent study by Kaila and colleagues[17] utilized B3LYP*-D3, highlighting its application in the field. Furthermore, there are other notable examples of the usage of B3LYP*-D2 and

B3LYP*-D3 in studies concerning various other systems[114-119].

Suppl. Note 12. Relative population of the S_0 isomers related to calculated energetics

Applying the fundamental equation $\Delta G° = -RT \ln K_{eq}$ (K = [A]/[B]) for a chemical equilibrium B ⇌ A allows us to estimate the relative population ratio $[S_0^A]/[S_0^B]$ or the percentage of S_0^A and S_0^B in an equilibrium state. In the case of the proposed isomerism S_0^B (closed-cubane) ⇌ S_0^A (open-cubane) mentioned in this study, an initial analysis reveals that with the calculated $\Delta G°$ of -0.6 kcal mol^{-1} for the doublet/αβαβ spin state depicted in Fig. 5, the relative population of $[S_0^A]/[S_0^B]$ would be approximately 1:0.36 (≈2.8) at room temperature. This estimation suggests a significant presence of S_0^B, which seems inconsistent with the results obtained from XFEL experiments indicating the absence of the closed-cubane structure in the S_0 state.[120] Nevertheless, it is important to highlight that attempting to accurately quantify the relative population using DFT methodology is not feasible due to the inherent limitations and approximations[96,98,121-124]. Therefore, imposing constraints between the relative population and calculated energetics is ill-advised and not recommended.

The aforementioned formula is commonly employed to estimate $\Delta G°$ by accurately measuring constituent concentrations in experimental studies, such as the work conducted by Messinger and colleagues on the driving force of the $S_3 \rightarrow S_0$ transition.[91] However, when using $\Delta G°$ obtained from DFT calculations to estimate the relative population of components (such as $[S_0^A]/[S_0^B]$ discussed here), it is crucial

to acknowledge the high sensitivity of the $[S_0^A]/[S_0^B]$ ratio to the value of $\Delta G°$. This sensitivity arises from the logarithmic nature of the relationship, meaning that even a slight variation in $\Delta G°$ (in kcal mol^{-1}) can result in a substantial (in fact, exponential) change in the estimated relative population. For instance, a slight decrease in $\Delta G°$ from 0.0 to -0.1, -0.5, -1.0, -2.0 would lead to a $[S_0^A]/[S_0^B]$ ratio changing from 1.0 to 1.2, 2.3, 5.4, 29.3, respectively. Conversely, a slight increase in $\Delta G°$ from 0.0 to 0.1, 0.5, 1.0, 2.0 would result in the $[S_0^A]/[S_0^B]$ ratio changing from 1.0 to 0.84, 0.43, 0.18, 0.03, respectively. Given the expected and inevitable few kcal mol^{-1} error due to the precision limitations of current DFT methods, caution should be exercised when interpreting the value of $[S_0^A]/[S_0^B]$ estimated from $\Delta G°$. Moreover, it is important to note that different choices of models and computational methods (as illustrated in *Suppl. Figs.* 11 and 13, considering various functionals) may introduce slight variations in calculated energetics. As discussed earlier, these variations can lead to significant uncertainties in terms of relative populations.

The above demonstration is also reflected in the literatures regarding the well-known open-closed isomerization in both the S_2 and $S_2Y_Z^\bullet$ states. Pantazis et al.[125] show that the relative energy between the S_2^A and S_2^B conformations is approximately 1 kcal mol^{-1}, specifically 1.92 kcal mol^{-1} according to B3LYP and 0.42 kcal mol^{-1} according to TPSSH-D3. This corresponds to $[S_2^B]/[S_2^A]$ ratios of 0.04 and 0.71, respectively. Several other studies, including those by Bovi et al.[50], Siegbahn[111], Ugur et al.[126], Boussac et al.[127], Vinyard et al.[128], Isobe et al.[30], Saitow et al.[129] reported ΔG values ranging from 1.1 to 2.0 kcal mol^{-1}, with the smallest $[S_2^B]/[S_2^A]$

ratio being 0.03. In the case of the $S_2Y_Z^{\bullet}$ state, the relative stability is reversed, as noted by Lohmiller et al.[71] and Narzi et al.[49]. The former reported a 1.3 kcal mol^{-1} higher energy for $S_2^BY_Z^{\bullet}$ than for $S_2^AY_Z^{\bullet}$, while the latter instead reported 2.6 kcal mol^{-1} lower energy for $S_2^BY_Z^{\bullet}$ than for $S_2^AY_Z^{\bullet}$, indicating a difference of 3.9 kcal mol^{-1} and a range of the ratio $[S_2^BY_Z^{\bullet}]/[S_2^AY_Z^{\bullet}]$ from 0.11 to 80.7. It is evident that despite the minor thermodynamic energy differences within a few kcal mol^{-1}, the relative population in the $S_2Y_Z^{\bullet}$ state should be regarded as quite uncertain and highly dependent on the various DFT models and methods used. Recently, a similar phenomenon was recently reported for the isomerism in the S_1 state[37], where the authors also emphasized this issue. Consequently, it is important to consider the implications of small errors in Gibbs free energy differences when attempting to establish a direct connection to substance population or percentage. However, it is crucial to note that these small errors in energetic estimates typically fall within a few kcal mol^{-1} and do not significantly impact the qualitative determination of the feasibility of the proposed isomerism.

Suppl. Note 13. Validity of the closed-cubane structure in the catalytic cycle

The notion of the closed-cubane structure of the OEC cluster was initially proposed by Pantazis et al.[125] (as well as Isobe et al.[30]) to provide an explanation for the higher spin (S=5/2) EPR signal at g=4.1 observed in the S_2 state. This closed-cubane structure can undergo a μ-O5 shift, leading to the interconversion of the S_2 state to a low-spin (S=1/2) multiline signal observed at g=2. Additionally, the concept of the closed-cubane structure has been extended to include the S_1 state[37], S_2-S_3

transition[6,17,38,42,48-50,76,128], S_3[34-36,130], S_3Y_Z•[131] and S_4[6,20,21,132] states, and in water exchange mechanisms[5,6,33,43,133]. Nevertheless, there are two additional theoretical models that could potentially explain the presence of the high-spin (S=5/2) S2 state within the context of open-cubane structures. These models include the μ-O4 protonation scheme proposed by the O'Malley group[44] and the binding of OH^- to Mn1(IV) as suggested by Siegbahn[111] and Pushkar et al.[46], respectively. All of these distinct models can be justified and explained under different conditions, treatments, and scenarios, making them viable contenders. However, several factors support the preference for the closed-cubane model. Firstly, the closed-cubane model aligns well with available spectroscopic findings, including constraints derived from extended X-ray absorption fine structure (EXAFS) measurements, exchange coupling constants (J_{ij}), and isotropic ^{55}Mn hyperfine constants[125]. Secondly, EPR studies conducted by Mino and colleagues have confirmed the presence of Mn(III) at the dangler position in the high-spin S_2 configuration[134,135]. This observation suggests that there is a distribution of oxidation states, specifically Mn1(IV)Mn2(IV)Mn3(IV)Mn4(III), which indicates a valence rearrangement resembling the open-closed isomerism. Additionally, it is worth noting that the low- and high-spin forms of the system exhibit nearly iso-energetic behavior, as evidenced by their coexistence at very low temperatures[127,136-138]. Experimental estimations of the energy difference between these forms indicate a value of approximately (0.7 ± 0.1) kcal mol^{-1}. Notably, only the open/closed-cubane model offers a computed energy difference of a similar magnitude, ranging from 1-2 kcal mol^{-1} [49,50,125-129,139,140], whereas the other two

models yield values of approximately 7 kcal mol-1[111] and 10 kcal mol^{-1} [44], respectively. Furthermore, the mechanistic rationale behind the kinetics of substrate-water exchange supports the notion that closed-cubane forms serve as essential intermediates[5,6,33,43,133]. Fifthly, a recent theoretical study by Saito et al.[141] supports the closed-cubane S_2 conformation (with W1=OH^-) for explaining the g=4.1 signal, which has later been experimentally confirmed by Kosaki and Mino using Q-band pulse EPR spectroscopy[142]. Until now, the closed-cubane model has not been observed by XFEL crystallization techniques in the studies by the two major groups at Berkeley and Okayama[1,3,94,120,143-147]. Nevertheless, it is important to approach these findings with caution as they do not definitively rule out the possibility of isomerism and the potential presence of closed-cubane forms. While the time-resolved structures offer compelling evidence regarding the fundamental structural characteristics of stable intermediates, it is crucial to recognize that the information provided may not be strictly limiting but rather inclusive of structural variations. Several reflections and references highlight the existing controversies and uncertainties regarding the true nature of these structures. Cox et al.[148], for instance, have emphasized that it remains unclear whether a particular state, capable of existing in different conformations, would exhibit all of these forms under the specific experimental conditions employed. Despite the absence of direct observation of the closed-cubane structure during the $S_2 \rightarrow S_3$ transition, Ibrahim et al.[143] have put forth speculations suggesting its potential existence. They proposed that the closed-cubane structure might have formed but decayed before their first time point of detection (at 50 μs), or that its short-lived

nature could be attributed to rapid formation and decay kinetics. Another possibility is that its fraction, for various reasons, remains below 10% at each measured time point. It is important to note that the lack of detection of an isomer does not necessarily imply its complete absence as potential intermediates. Considering the aforementioned factors, the closed-cubane model remains a valid structural model and could potentially play significant roles in different stages of the catalytic cycle.

Suppl. Note 14. Complementary explanation for the truncated model construction

Based on the truncated model including Asp61, our attempt to localize an open-cubane structure and the corresponding TS from the closed-cubane structure after water dissociation was not successful. Our analysis is that Asp61 forms strong double hydrogen bonds with W1 and W7 (ligands of Mn4), which hinders contraction of the cluster (decreasing the Mn1-Mn4 distance) in an open-cubane structure. On the other side, while W1 releases one H^+ via Asp61, keeping Asp61 in its original position in the MEP calculations is inappropriate, because it would become quite spatially flexible upon protonation. This can be observed in the classical MD simulation by Rivalta et al.[81] and the QM/MM MD simulation by Narzi et al.[49] Thus, it is supposed that after W1 deprotonation, Asp61 should be largely deviated from the current position shown in Im2, but this dynamics could not be reflected in a finite MD structure model with backbone constraints. Retaining Asp61, without representing its correct position in the S_0 state, would cause artifacts instead. While the functional significance of Asp61 as a second-sphere ligand for the OEC cannot be overlooked,

and its protonation state[23,93,149], along with W1, could potentially impact the various conformations and their corresponding energetics of the Mn4Ca cluster[29], it is deemed appropriate to exclude it from the model system for the specific investigation here. This decision is based on the following reasons. First and foremost, the remote location of Asp61 suggests that its influence on the reaction energetics under consideration would be minimal, given that these reactions primarily occur within the cluster itself. Secondly, the molecular mechanism by which the protonation state of Asp61-W1 affects the S2 isomers is closely tied to the valence alteration of Mn4[29]. W1(H_2O) coordinated to Mn4(III) (in a closed-cubane S_2 structure) has a higher pK_a and thus remains as a neutral W1(H_2O)...Asp61, while the pK_a of water ligand coordinated to Mn4(IV) (in an open-cubane S_2 structure) is lower resulting in W1(OH^-)...H^+Asp61. In contrast, for the S_0 state investigated in this study, there is no valence change on any Mn center during the isomerization that would affect the pK_a of the water ligands. All these weaken the necessity to involve Asp61 in the truncated DFT model for the subsequent MEP searches after W1 deprotonation. Analogous considerations have previously been adopted in a cluster model by Retegan et al. in a study of the S_2-S_3 transition[38]. However, for the S_0 isomerism, in order to verify its validity in the presence of Asp61 (in its correct position in the S_0 state), additional calculations including Asp61 in the model (222 atoms, net charge 0) were carried out by using the 3F X-ray free electron laser (XFEL) data (PDB ID: 6DHP) with the S_0 state as the major population (after removing the mixed population of S_3 state) (Suppl. Table 6 and Suppl. Figs. 10-11).

Suppl. Note 15. General scheme of IRC and comparison with NEB

The IRC procedure[150] is well established (being developed for more than 40 years) with many examples and analyses in the literatures. It is a widely used method for modelling the minimum energy pathway (MEP) in chemical reactions, which is defined as the steepest-descent path on the potential energy surface (PES) from the transition states (TS) down towards a local minimum, i.e. reactant and/or product. This can be done, in principle, in any coordinate system. An IRC path is defined similarly but in particular using mass-weighted coordinates and employing Hessian matrix to predict the downhill direction. This means that instead of being guided by the steepest descent direction, it follows the maximum instantaneous acceleration. In the present study, TSs were located by the Berny algorithm[151] and the transit-guided quasi-Newton (STQN) method[152] embedded in Gaussian. The energy profile is obtained, as well as the length and curvature properties of the path, providing the basic quantities for an analysis of the reaction path. Practically, to perform an IRC calculation, several steps are typically involved: 1) the TS geometry is optimized to identify the stationary point on the PES; 2) the Hessian matrix is calculated at the TS geometry to obtain the vibrational frequencies and normal modes; 3) the atoms are displaced along the imaginary vibrational mode associated with the lowest frequency to initiate the IRC calculation; 4) the differential equations of motions are numerically integrated to determine the MEP, and integration is typically carried out using algorithms such as the Hessian-based predictor-corrector integrator[153]; 5) the calculated MEP can be visualized using various software tools to gain insights into the

reaction mechanism.

Instead of first optimizing a TS by surface walking[154] and calculating a reaction path afterwards, both can be obtained simultaneously from the chain-of-states methods, such as the nudged elastic band (NEB) approach[155], which was developed much later than IRC. NEB optimizes a series of intermediate images positioned between the initial and final states of the reaction. These images are evenly spaced along the reaction path and are optimized to find the lowest energy configurations while maintaining a fixed separation from their neighboring images. Spring forces are introduced between adjacent images to simulate the elastic behavior of a band. These spring forces act to guide the atoms along the reaction path and prevent excessive distortion. The magnitude of the spring forces is typically set to zero for the initial and final states. NEB provides the tangent forces acting on each image, which represent the direction of maximum energy change along the reaction path. To ensure that the atoms move along the MEP rather than being influenced solely by the tangent forces, the NEB forces are obtained by projecting the component of the forces that is perpendicular to the band. By optimizing the positions of the intermediate images while considering the spring forces and projecting the forces, NEB provides a method to explore and determine the MEP and transition states on the potential energy surface.

While IRC is more widely used in the molecular modelling community, NEB seems more popular in the solid state and surface physics/chemistry. IRC and NEB are both useful tools, but we to make some practical considerations. Below some general advantages and limitations for IRC and NEB are summarized. IRC

calculations provide a detailed analysis of the reaction pathway along the MEP and allow for the visualization and understanding of the bond-breaking and bond-forming events during a reaction. IRC calculations are conceptually simpler and are generally computationally faster as compared to NEB because IRC follows the steepest descent path along the MEP (as long as an accurate Hessian for the TS is given initially) without the need for interpolation schemes or optimization of intermediate images. The accuracy of the IRC results can be sensitive to the initial guess of the TS geometry. If the initial guess is not close to the 'true' transition state, the IRC path may not accurately represent the reaction pathway, and therefore it is crucial to make reasonable initial guesses to obtain reliable results. In complex systems, there may be multiple possible reaction pathways and transition states. IRC calculations may not effectively explore these alternative reaction pathways, as they are limited to following the steepest descent path from the starting geometry. The main advantage of NEB is that only gradients are required in regular optimizations or surface scans (no exact Hessian is required) but unlike surface scans, the method converges to the MEP and allows convenient saddle-point optimization in the same computational job. Surface scans are also strongly biased towards the choice of the reaction coordinates and can often end up far from the MEP, while NEB has no such bias (there is a small bias towards the initial interpolated path). The accuracy of NEB results can be sensitive to the initial guess for the positions of the intermediate images along the reaction path. If the initial guess is far from the true path, it may require more iterations and adjustments to accurately converge on the MEP. This sensitivity

highlights the importance of making reasonable initial guesses for the intermediate states. The choice of interpolation scheme can affect the accuracy and convergence of the NEB calculations. Different interpolation schemes, such as linear or higher-order polynomial interpolation, may yield different results. The selection of an appropriate interpolation scheme is crucial for obtaining reliable and accurate results. NEB calculations can be computationally expensive, especially for larger and more complex systems. The optimization of multiple images along the reaction path requires additional computational resources, including time and memory. The computational cost may limit the size and complexity of the systems that can be studied using NEB.

Suppl. Note 16. Relevance to femtosecond X-ray crystallography during O_2 formation

Bhowmick et al.[1] observed that from 1,200 to 4,000 μs several structural changes occur, and most of these changes are indicative of O_2 release and/or water insertion. This indicates that the O_2 release and refilling of the cluster by bulk water and resetting of the catalytic center occur over an extended timescale. Within this period, 1,200 and 2,000 μs are the two essential timepoints that are closely related to our work. According to their identification, the 1,200 μs snapshot signifies the onset of O_2 evolution; the 2,000 μs snapshot, without Ox on the electron density omit map, indicates completion of binding of a water that refills the vacant site formed by O_2 release. On this basis, the process we suggest in the present study should in principle transiently resides between these two timepoints, however covering a very short

period because of the picosecond timescale for water insertion and nanosecond timescale for the subsequent closed-to-open- cubane transformation of the cluster. It is understandable that the ultrafast conversion cannot be captured by the XFEL crystallography with hundreds of microseconds as the interval. According to Bhowmick et al., additional distances changes between Y_Z and His190 are observed between 2,000 and 4,000 μs, which may be due to the rearrangement of the HB network related to the last proton release but are not well understood currently. In our proposal, H^+ is released from W1 (as the new W2) to lumen *via* Asp61 in Im2, and this is assumed to be the second proton release during the S_3-S_0 transition. After gated by Asp61, the released H^+ would pass by substantial water and protein residue groups located in the proton channel of PSII which may cause pronounced variations in rearrangement of the HB network (including water-water, protein-protein, and water-protein interactions) coupled to protein dynamics[156] and possibly other unknown structural changes, together responsible for the extended timescale between 2,000 and 4,000 μs. For example, the increase of the Y_Z–D1-H190, Ca–D1-E189 and Mn4–O5 distances and decrease of the Mn1–Mn4 distance were observed. There could be chances of seeing intermediates during the extended timescale for future crystallographic snapshot data. Thus, we clarify that neither we suggest the resetting of the catalytic center is at picosecond timescale nor our suggested process covers the whole period from 1,200 to 4,000 μs; but rather the mechanism/progression revealed by our MD simulations and MEP calculations represents only an ultrashort phase (from picosecond to nanosecond timescale) embedded in the timepoints between

1,200 (precisely some timepoint after this when O_2 has been released) and 2,000 μs. Therefore, our suggestion does not conflict with Bhowmick et al.'s observation. Besides, it is noted that in Bhowmick et al.'s definition, the final S_0 state is assigned to 3F(200 ms); however, by the 2,000 μs timepoint after 3F, the O5 omit map density is restored considerably compared with the S_3 and S_0 states, which indicates that water insertion after O_2 release has occurred, albeit not yet the final S_0 state with fully restored electron densities and other qualified indicators. In our modelling and definition, the final S_0 state (i.e. the S_0^A state) is reached as long as the open-cubane structure is formed within the Mn_4CaO_5 cluster, which should correspond to a timepoint close to 2,000 μs. Thus, there is a semantic discrepancy regarding the definition of the S_0 state between Bhowmick et al.'s and ours, but both share the most important point that O5 has been restored between Mn3 and Mn4 as the basic feature of the cluster. Since our model and simulations cannot and do not aim to represent other structural changes after 2,000 μs toward the S_0(3F(200 ms)) state, the above-mentioned caution should be taken when compared to the experimental findings regarding the extended timescale.

Suppl. Note 17. Roles of specific water molecules and hydrogen-bond interaction

The single-electron—multi-proton transfer event proposed by Greife et al.[2] includes a single electron transfer from Ox to Y_Z●, concerted with multiple proton transfers from Ox(OH^-) to W3(H_2O), W3 to W2(OH^-) and W1(H_2O) to Asp61(COO^-). It is the slowest step in photosynthetic O_2 formation with a moderate energetic barrier and marked entropic slowdown, leading to the oxygen-radical S_4 state for fast O-O

bonding and O_2 release. Obviously, the specific location of water molecules and the hydrogen-bond (HB) interactions between them play a very important role for facilitating the critical step of Mn(IV)-O● formation. The multi-proton transfer would not take place without the water molecules being present in a HB network located along the route. Greife et al. also tested the cases of electron transfer from Ox without proton movements, but this would significantly destabilize the system indicating the importance of simultaneous proton transfer in the HB network. The HB interactions of Ox-W3 (formation after Ox-Glu189 breakage), W3-W2, and W1-Asp61 are essentially the effective ones. Since the free energy barrier from the S_3Y_Z● to S_4 transition was determined to be 13.6 kcal mol^{-1} as the kinetic bottleneck, the concerted proton transfer cannot be captured in a QM/MM MD simulation in tens of picoseconds, and minimum energy path (MEP) calculations (using the NEB method) were employed to study the proton transfer and determine the barrier (accompanied by electron transfer). In our present study, the specific locations of water molecules and HB interactions are also crucial for the resetting process of the Mn_4CaO_5 cluster. Specifically, our simulations show the crucial roles of the water molecules at least closely around the cluster, i.e. W1, W2(OH^-), W3, W5, W6, W7 and W8 and the HB interactions of W5-W6, W3-W5, W3-W2 and W7-W8. Due to the limitation of the model size, HB interactions between the water molecules locating further away (outside the model, with minor effect for the cluster resetting process) cannot be reflected but their motions driven by the HB interactions can also be expected. Among them, here we emphasize the particularly crucial role of the Ca-bound W3 and the

Mn4-bound W2 and the HB interaction between them in the cluster resetting process. During the process of W3 moving to the cavity, it forms strong HB interaction with W2 which is the partial reason (the other reason being Mn charge distribution) for W3 binding to Mn1 instead of Mn4, i.e. binding to Mn1 has results in an energetically more favorable HB orientation to W2, and W2 rotates around Mn4 toward W3 which to some degree hinders W3 approaching to Mn4. After Im1 formation, the W3-W2 HB interaction appears more important because the short and strong HB interaction leads to a low-barrier (or even barrierless) proton transfer between them which facilitates the following structural evolution. W3 spontaneously deprotonates to W2 which causes W3 to approach Mn3 (creating the closed-cubane structure) and the 'pivot/carousel'-like ligand reorganization around the dangler Mn4, together with W2 rotation after its protonation. In the meanwhile, the cluster is expanded seen from the elongated Mn1-Mn4 distance and larger ∠Mn3-O4-Mn4, and the resultant geometric change of the Mn4 coordination has created an empty coordination layer toward W7 from the O4 channel. W7 binding to Mn4 occurs immediately after (or almost synchronous with) W2 protonation because of further rotations of W2 and W1 and decreased structural *trans* effect on Mn4. The HB interaction of W3-W5 makes W5 occupy the original coordination of W3 on Ca. Besides, W6 and W8 motions pulled by the HB interactions of W5-W6 and W7-W8 (and similarly others outside the model) are indispensable for recovery of the surrounding water environment of the OEC. Different from the single-electron multi-proton transfer with a moderate barrier observed for Greife et al., the present case does not involve electron transfer because

there is no electron hole on Y_Z or any radical on the ligands; the present case only involve one proton transfer from W3(H_2O) to W2(OH^-), which is considered barrierless (or almost) since it is observed to take place spontaneously on the picosecond timescale (we surmise the Ox-H deprotonation in Greife et al.'s case should be more demanding than H_2O deprotonation). This is also consistent with the fast kinetics for the S_4-S_0 transition post to O-O bond formation.

Suppl. Note 18. Comparison to previous reports

Previous to our present study, Capone et al.[47] also looked into the mechanism of oxygen evolution and Mn_4CaO_5 cluster restoration by MD simulations, in which the major contributions mainly include two points: 1) confirmation of the Ca-bound W3, instead of Mn4-bound W2, as the water molecule inserting into the cavity formed by the O_2 release; 2) validation of the two-step (stepwise) mechanism for O_2 release and water (W3) insertion (against the concerted mechanism[63]). For our present study, the observation that W3 is the inserted water molecule is in good agreement, and our starting structure with the complete removal of the O_2 molecule is also consistent, also in line with Siegbahn's previous results[8]. For reconstitution of the Mn_4CaO_5 cluster, while Capone et al. assume W3 (along with deprotonation to W2) would directly move to the bridge position between Mn3 and Mn4 and form the open-cubane structure of the S_0 state, we instead propose a different pathway for how W3 should enter the cavity, i.e. W3 binding first to Mn1 for formation of the closed-cubane intermediates along with W3 deprotonation to W2, which triggers the 'pivot/carousel'-like ligand reorganization around Mn4 and W7 coordination to Mn4,

and then the open-cubane structure of the S_0 state will form after W2(H_2O) dissociation and W3(OH^-) transfer to Mn4. Regarding the MD methodology, generally, Capone et al. used QM(DFT)/MM MD for 10 ps with a small QM size, while we used *ab initio* (DFT) MD with a larger QM size for 30 ps which observes the spontaneous W3 binding. Besides, there are some notable differences in the computational details. For example, Capone et al. used Nos-Hoover thermostat while we used Bussi-Parrinello Langevin thermostat; Capone et al. used PBE+U/DZVP-MOLOPT-SR-GTH basis set, which is a plane-wave method (although Gaussian orbitals are added as an adjunct), while we used standard Gaussian basis set UB3LYP/LanL2DZ/6-31G*/3-21G; etc. We are not so sure if the methodological differences may have large effects on the results (without recommending or criticizing one over the other), but Capone et al. did not test the possibility proposed here and just followed the more straightforward movement of W3 (also for W2) coordinating Mn3/Mn4 in their directed MEP calculations. In their MD simulation within 10 ps, they indeed found the predisposition of W3 moving near the vicinity of the cavity, but it is uncertain whether the further movement of W3 onto a specific Mn (toward Mn1 or Mn4) could be observed if the simulation time were extended. Finally, we emphasize equal importance for the two plausible routes, without excluding the possibility of the old scenario, and further comparative studies are needed. However, while the target structure is the same, we present possible intermediates in closed-cubane structures *en* route to reach the S_0 state, which are important for understanding the structural flexibility prevailing in the cycle.

II. Supplementary Tables

Suppl. Table 1. Gibbs free energies of $Im0^{-O_2}$, Im1 and Im2 for the octet/αααβ and doublet/αβαβ spin states computed at the level of B3LYP*-D3(BJ)/SDD/cc-pvtz(-*f*)/SMD(ε=6.0)//B3LYP*-D3(BJ)/LanL2DZ/6-31G*.

Spin states		Gibbs free energies in a.u. (Relative Gibbs free energies in kcal mol^{-1})		
		$Im0^{-O_2}$	Im1	Im2
Octet	αααβ	-9970.4418 (0.8)	-9970.4556 (-7.9)	-9970.4898 (-29.4)
Doublet	αβαβ	-9970.4430 (0.0)	-9970.4582 (-9.5)	-9970.4922 (-30.9)

Spin states			Mulliken spin populations		
			$Im0^{-O_2}$	Im1	Im2
Octet	α	Mn1	3.86	3.89	3.86
	α	Mn2	3.04	3.03	2.99
	α	Mn3	3.79	3.79	3.80
	β	Mn4	-3.80	3.79	-3.80
Doublet	α	Mn1	3.85	3.91	3.87
	β	Mn2	-2.96	-2.94	-2.91
	α	Mn3	3.76	3.78	3.81
	β	Mn4	-3.80	-3.79	-3.80

Suppl. Table 2. Reaction energetics of W1(H_2O) deprotonation to Asp61 for the octet/αααβ and doublet/αβαβ spin states computed at the level of B3LYP*-D3(BJ)/SDD/cc-pvtz(-*f*)/SMD(ε=6.0)// B3LYP*-D3(BJ)/LanL2DZ/6-31G*.

**Spin states**		**Electronic energies in a.u. (Relative electronic energies in kcal mol^{-1})**		
		**W1(H_2O)-Asp61**	**TS(PT)**	**W1(OH^-)-Asp61-H**
**Octet**	**αααβ**	-6039.2899 (1.9)	-6039.2884 (2.8)	-6039.2907 (1.4)
**Doublet**	**αβαβ**	-6039.2929 (0.0)	-6039.2915 (0.9)	-6039.2938 (-0.6)
**Spin states**		**Gibbs free energies in a.u. (Relative Gibbs free energies in kcal mol^{-1})**		
		**W1(H_2O)-Asp61**	**TS*(PT)**	**W1(OH^-)-Asp61-H**
**Octet**	**αααβ**	-6037.6776 (0.9)	-6037.6777 (0.8)	-6037.6772 (1.1)
**Doublet**	**αβαβ**	-6037.6790 (0.0)	-6037.6791 (-0.1)	-6037.6786 (0.3)

*For a proton transfer (PT) reaction with a very low TS barrier in electronic energy, it is normal that the Gibbs energy of TS may be even (slightly) lower than the adjacent stationary structures because of the little contribution to ZPE. This indicates the deprotonation process is quite facile. This phenomenon has also been reported in some literatures for other systems [157-160].

Suppl. Table 3. Gibbs free energies of pre-S_0(W2-bound), TS(Mn4-W2) and pre-S_0(W2-unbound) for the octet/αααβ and doublet/αβαβ spin states computed at the level of B3LYP*-D3(BJ)/SDD/cc-pvtz(-*f*)/SMD(ε=6.0)// B3LYP*-D3(BJ)/LanL2DZ/6-31G*.

Spin states		Gibbs free energies in a.u. (Relative Gibbs free energies in kcal mol^{-1})		
		**pre-S_0(W2-bound)**	**TS(Mn4-W2)**	**pre-S_0(W2-unbound)**
**Octet**	**αααβ**	-5769.3733 (0.9)	-5769.3668 (5.0)	-5769.3694 (3.4)
**Doublet**	**αβαβ**	-5769.3748 (0.0)	-5769.3684 (4.0)	-5769.3713 (2.2)

Spin states			Mulliken spin populations		
			**pre-S_0(W2-bound)**	**TS(Mn4-W2)**	**pre-S_0(W2-unbound)**
**Octet**	**α**	**Mn1**	3.87	3.88	3.89
	**α**	**Mn2**	3.01	3.01	3.01
	**α**	**Mn3**	3.81	3.80	3.81
	**β**	**Mn4**	-3.83	-3.82	-3.82
**Doublet**	**α**	**Mn1**	3.87	3.87	3.88
	**β**	**Mn2**	-2.91	-2.90	-2.90
	**α**	**Mn3**	3.80	3.81	3.82
	**β**	**Mn4**	-3.83	-3.82	-3.82

Suppl. Table 4. Gibbs free energies of S_0^B(closed-cubane), TS(Mn1-W3-Mn4) and S_0^A(open-cubane) for the octet/αααβ and doublet/αβαβ spin states computed at the level of B3LYP*-D3(BJ)/SDD/cc-pvtz(-*f*)/SMD(ε=6.0)// B3LYP*-D3(BJ)/LanL2DZ/6-31G*.

Spin states		Gibbs free energies in a.u. (Relative Gibbs free energies in kcal mol^{-1})		
		S_0^B(closed-cubane)	TS(Mn1-W3-Mn4)	S_0^A(open-cubane)
**Octet**	**αααβ**	-5692.9494 (1.2)	-5692.9423 (5.6)	-5692.9508 (-0.3)
**Doublet**	**αβαβ**	-5692.9513 (0.0)	-5692.9439 (4.6)	-5692.9522 (-0.6)

Spin states			Mulliken spin populations		
			S_0^B(closed-cubane)	TS(Mn1-W3-Mn4)	S_0^A(open-cubane)
**Octet**	**α**	**Mn1**	3.90	3.89	3.90
	**α**	**Mn2**	3.00	2.98	2.89
	**α**	**Mn3**	3.81	3.81	3.82
	**β**	**Mn4**	-3.87	-3.89	-3.90
**Doublet**	**α**	**Mn1**	3.89	3.88	3.88
	**β**	**Mn2**	-2.89	-2.89	-2.89
	**α**	**Mn3**	3.83	3.82	3.82
	**β**	**Mn4**	-3.87	-3.90	-3.90

Suppl. Table 5. Gibbs free energies of pre-S_0(W2-bound), TS(Mn4-W2) and pre-S_0(W2-unbound) for W2 dissociation, and S_0^B(closed-cubane), TS(Mn1-W3-Mn4) and S_0^A(closed-cubane) for the S_0 isomerism under the doublet/αβαβ spin state computed at the level of X-D3(BJ)/SDD/cc-pvtz(-*f*)/SMD(ε=6.0)//X-D3(BJ)/LanL2DZ/6-31G* by different GD3BJ-parameterized DFT functionals X; the pre-S_0(W2-bound) and S_0^B(closed-cubane) states are set as the zero references for the two cases, respectively, in each functional.

**Doublet/αβαβ**		**Gibbs free energies in a.u. (Relative Gibbs free energies in kcal mol^{-1})**					
**X (DFT functional)**		**B3LYP***	**B3LYP**	**B3PW91**	**BP86**	**TPSSTPSS**	**PBEPBE**
**W2 dissociation**	**pre-S_0(W2-bound)**	-5769.3748 (0.0)	-5772.5768 (0.0)	-5770.5787 (0.0)	-5772.9005 (0.0)	-5772.9450 (0.0)	-5766.1876 (0.0)
	**TS(Mn4-W2)**	-5769.3684 (4.0)	-5772.5703 (4.1)	-5770.5709 (4.9)	-5772.8940 (4.1)	-5772.9382 (4.3)	-5766.1822 (3.4)
	**pre-S_0(W2-unbound)**	-5769.3713 (2.2)	-5772.5728 (2.5)	-5770.5723 (4.0)	-5772.8951 (3.4)	-5772.9390 (3.8)	-5766.1838 (2.4)
**S_0 isomerism**	**S_0^B(closed-cubane)**	-5692.9513 (0.0)	-5696.1138 (0.0)	-5694.1474 (0.0)	-5696.4360 (0.0)	-5696.4850 (0.0)	-5689.8147 (0.0)
	**TS(Mn1-W3-Mn4)**	-5692.9439 (4.6)	-5696.1064 (4.6)	-5694.1414 (3.8)	-5696.4309 (3.2)	-5696.4794 (3.5)	-5689.8083 (4.0)
	**S_0^A(open-cubane)**	-5692.9522 (-0.6)	-5696.1142 (-0.3)	-5694.1474 (0.0)	-5696.4376 (-1.0)	-5696.4832 (1.1)	-5689.8114 (2.1)

Suppl. Table 6. Gibbs free energies of S_0^B(closed-cubane), TS, and S_0^A(closed-cubane) for the S_0 isomerism by using the model including D1-Asp61 truncated from 6DHP, under the doublet/αβαβ spin state computed at the level of X-D3(BJ)/SDD/cc-pvtz(-*f*)/SMD(ε=6.0)//X-D3(BJ)/LanL2DZ/6-31G* by different GD3BJ-parameterized DFT functionals X; the S_0^B(closed-cubane) state is set as the zero reference in each functional.

Doublet/αβαβ		Gibbs free energies in a.u. (Relative Gibbs free energies in kcal mol^{-1})					
X (DFT functional)		B3LYP*	B3LYP	B3PW91	BP86	TPSSTPSS	PBEPBE
S_0 isomerism	S_0^B(closed-cubane)	-6037.2207 (0.0)	-6040.5770 (0.0)	-6038.4722 (0.0)	-6040.9103 (0.0)	-6040.9781 (0.0)	-6033.8782 (0.0)
	TS(Mn1-O5H-Mn4)	-6037.2160 (2.9)	-6040.5727 (2.7)	-6038.4677 (2.8)	-6040.9047 (3.5)	-6040.9741 (2.5)	-6033.8733 (3.1)
	S_0^A(open-cubane)	-6037.2203 (0.3)	-6040.5768 (0.1)	-6038.4710 (0.8)	-6040.9086 (1.1)	-6040.9775 (0.4)	-6033.8780 (0.1)

III. Supplementary Figures

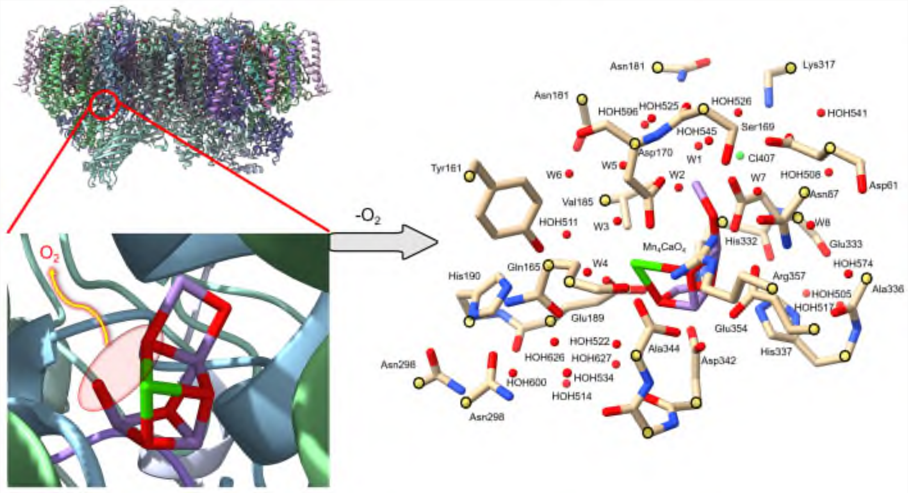

Suppl. Fig. 1. Model exhibition for MD. The initial BO-AIMD model for water insertion dynamics truncated from 6W1V, after removal of O5 and Ox for O_2 release (circled in red shadow on the left). The names for all the included residues and waters are also labelled. Protonation states are not shown for clarity. The small yellow circles on the right denote the fixed α-carbon atoms.

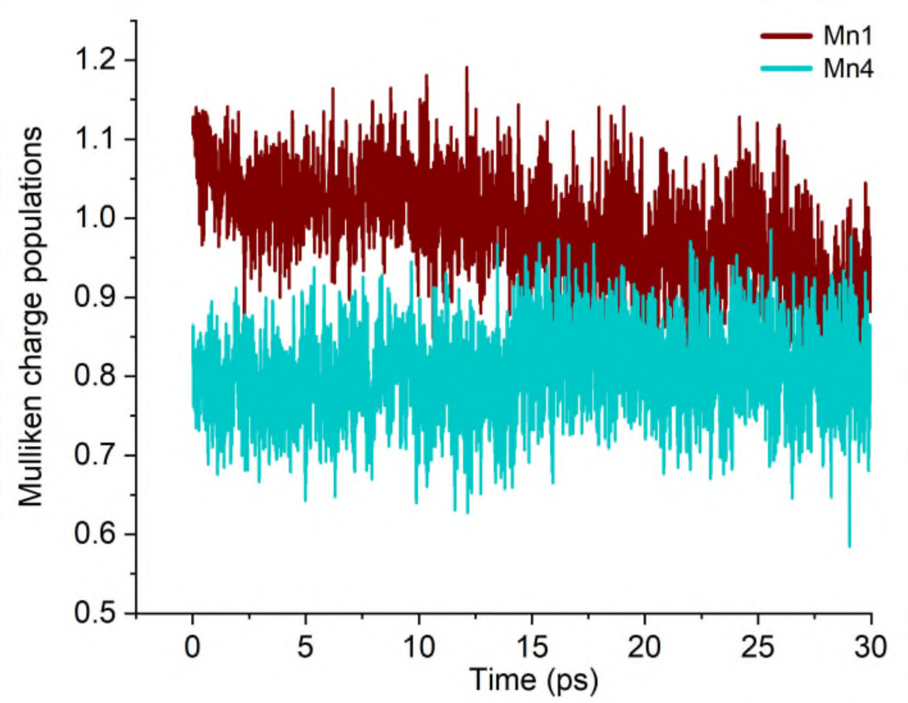

Suppl. Fig. 2. Charge populations during MD. Distributions of Mn Mulliken charge populations with time evolution along the BO-AIMD simulation trajectory for the octet/αααβ spin state.

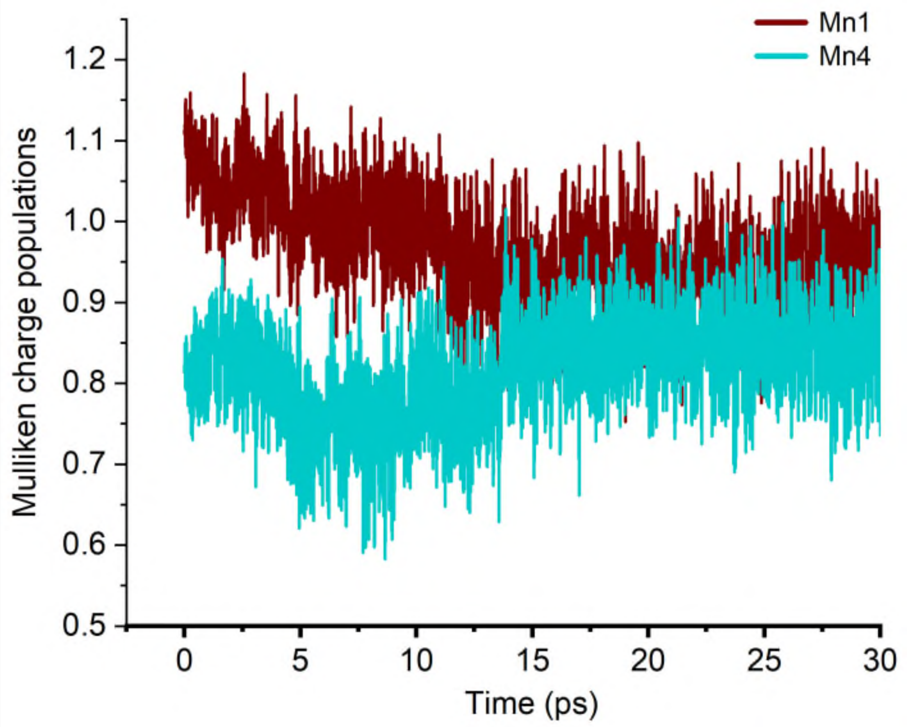

Suppl. Fig. 3. Charge populations during MD. Distributions of Mn Mulliken charge populations with time evolution along the BO-AIMD simulation trajectory for the doublet/αβαβ spin state.

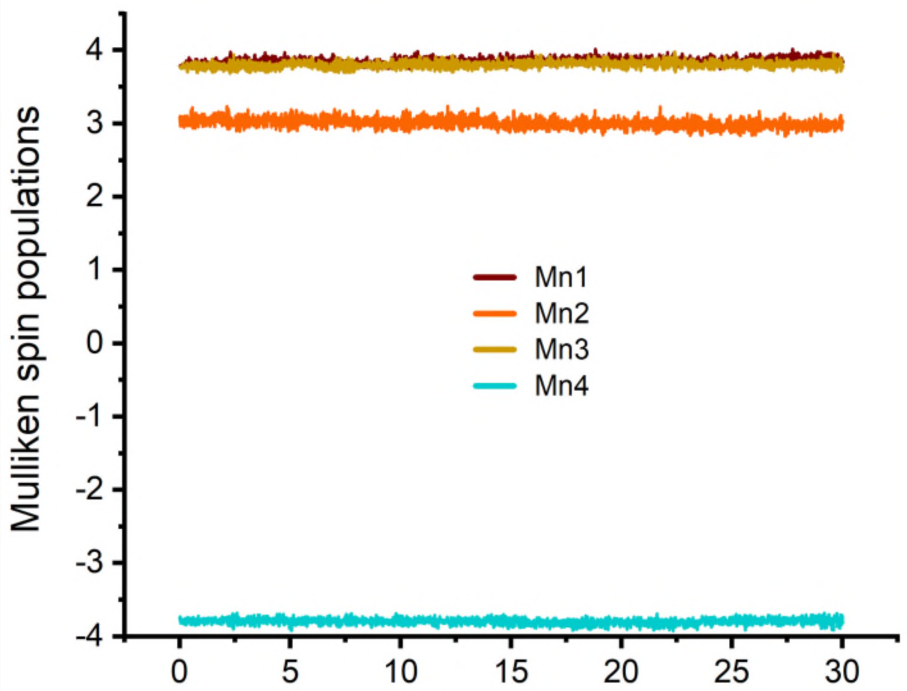

Suppl. Fig. 4. Spin populations during MD. Distributions of Mn Mulliken spin populations with time evolution along the BO-AIMD simulation trajectory for the octet/αααβ spin state.

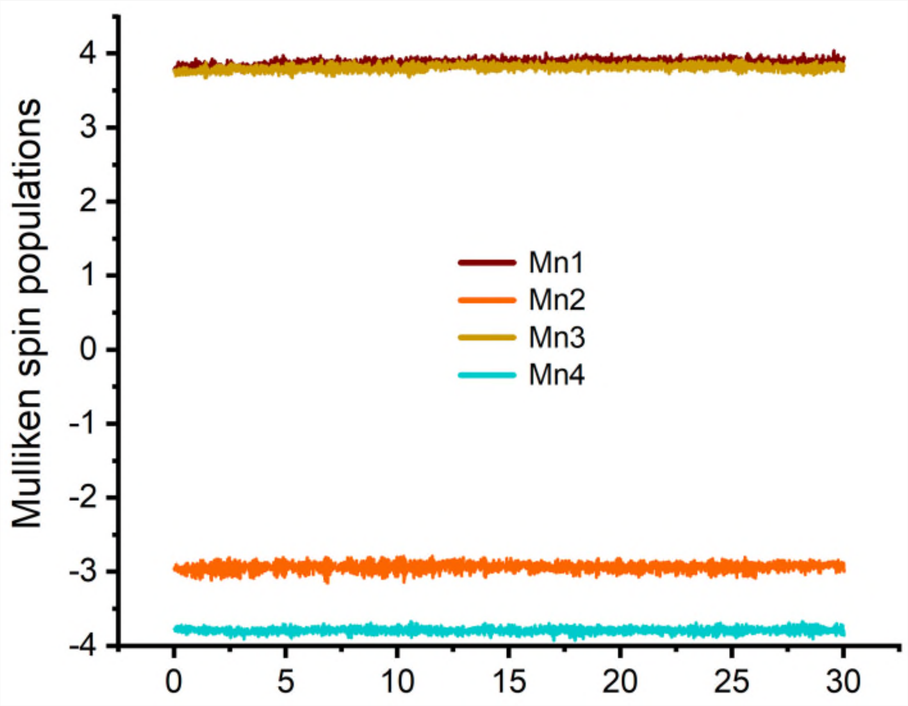

Suppl. Fig. 5. Spin populations during MD. Distributions of Mn Mulliken spin populations with time evolution along the BO-AIMD simulation trajectory for the doublet/αβαβ spin state.

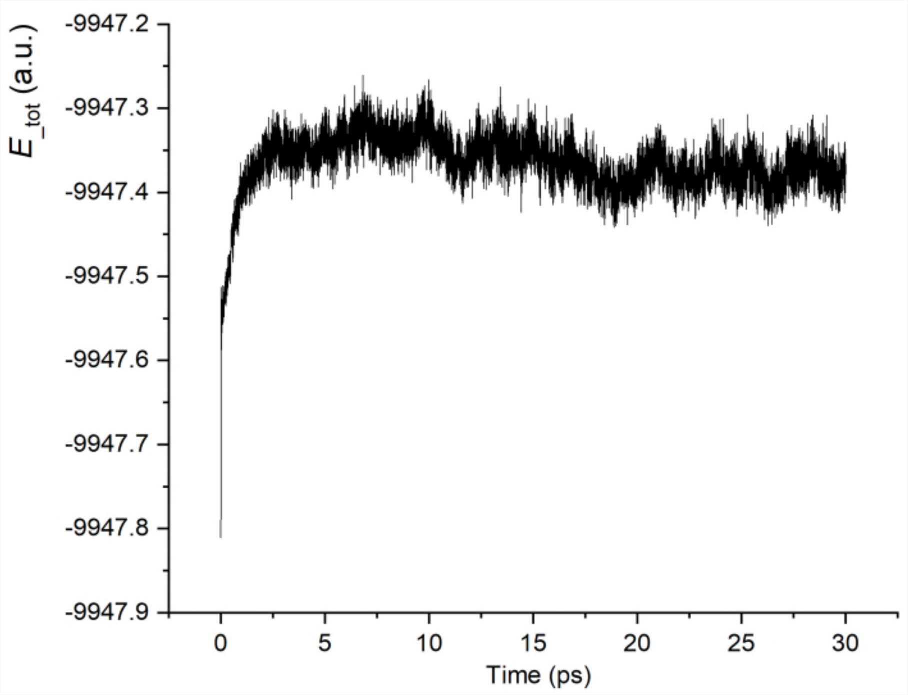

Suppl. Fig. 6. Total energy changes during MD. Distributions of the total energy $E_{\_tot}$ with time evolution along the BO-AIMD simulation trajectory for the octet/αααβ spin state. The initial energy rise in $E_{\_tot}$ is due to the fully optimized starting structure (with backbone constraints), similarly for Suppl. Fig. 7.

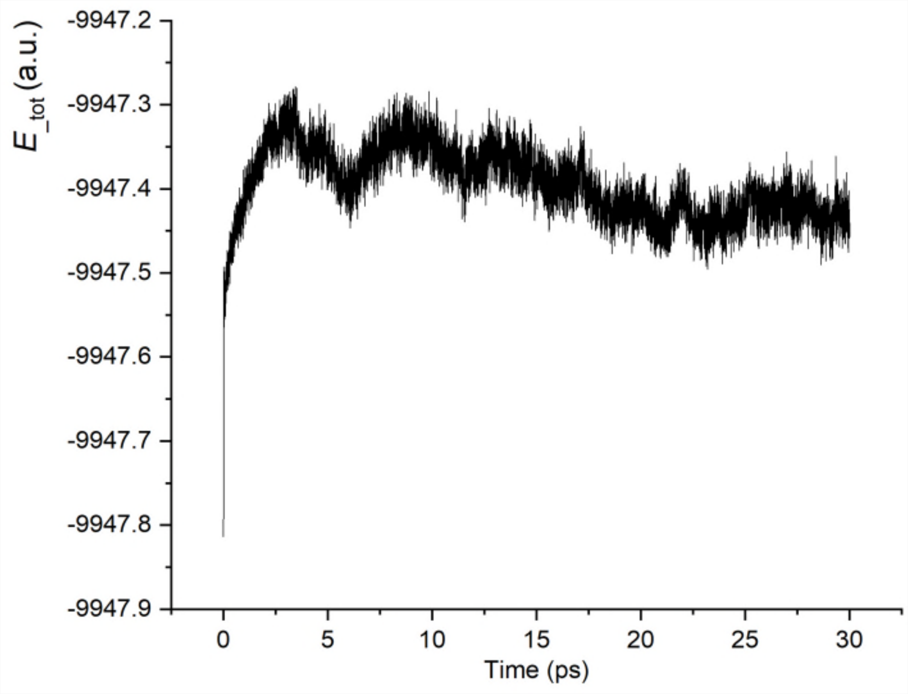

Suppl. Fig. 7. Total energy changes during MD. Distributions of the total energy $E_{\_tot}$ with time evolution along the BO-AIMD simulation trajectory for the doublet/αβαβ spin state.

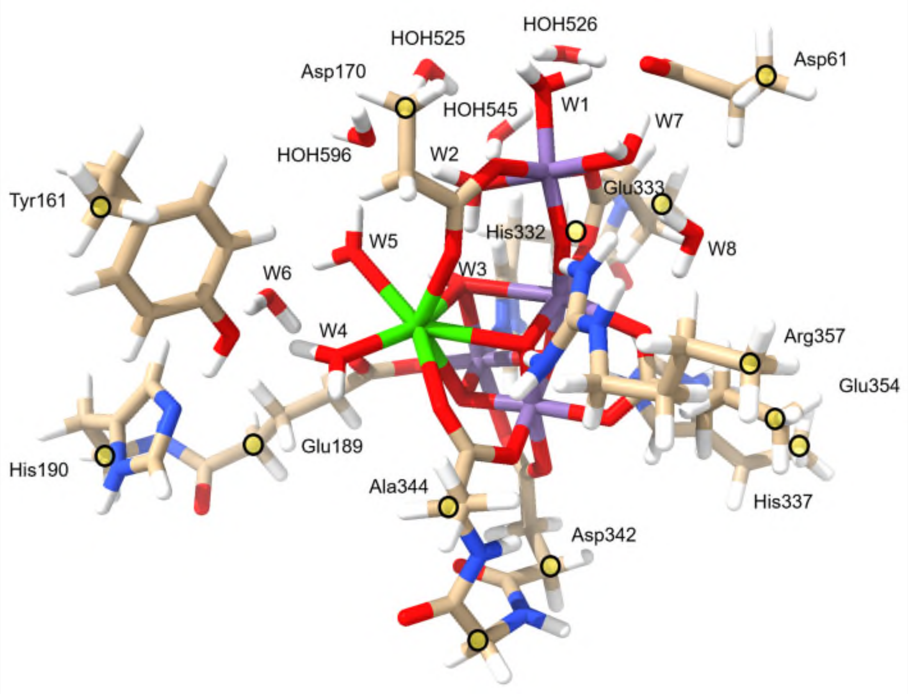

Suppl. Fig. 8. Model exhibition for MEP. The truncated DFT model for W1 deprotonation based on the last snapshot from the BO-AIMD simulation. The small yellow circles on the right denote the fixed α-carbon atoms.

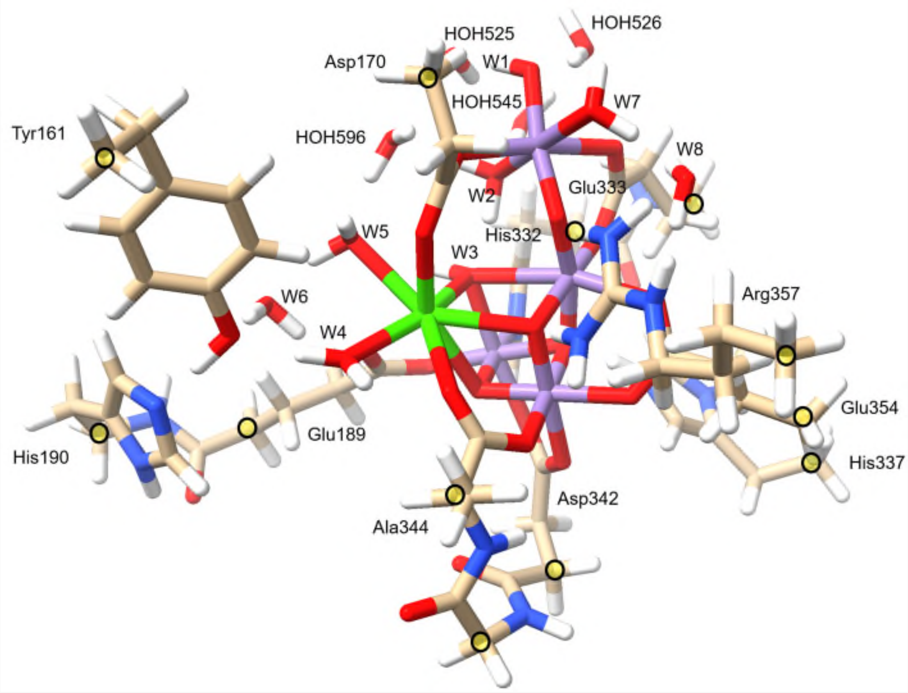

Suppl. Fig. 9. Model exhibition for MEP. The truncated DFT model for W2 dissociation after W1 deprotonation. The small yellow circles on the right denote the fixed α-carbon atoms.

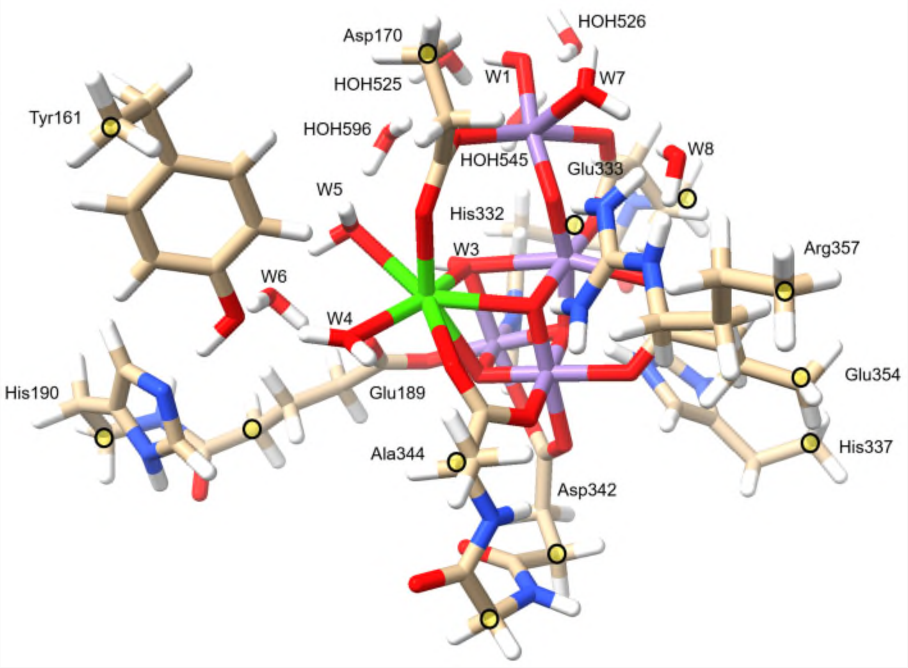

Suppl. Fig. 10. Model exhibition for MEP. The truncated DFT model for W3 shift after W2 dissociation. The small yellow circles on the right denote the fixed α-carbon atoms.

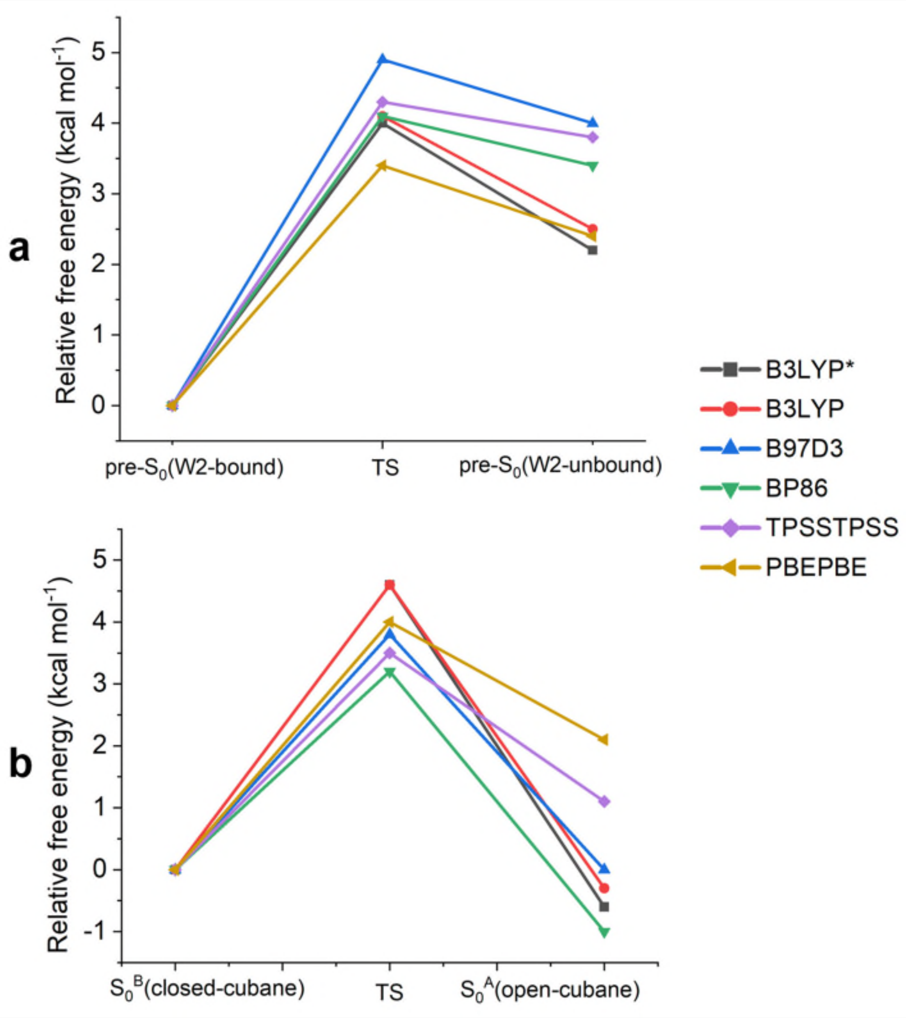

Suppl. Fig. 11. Functional sensitivity test. Functional dependence of the reaction energetics for **a** water dissociation in the pre-S_0 state and **b** the subsequent structural isomerism in the S_0 state under the doublet/αβαβ spin state computed at the level of X-D3(BJ)/SDD/cc-pvtz(-*f*)/SMD(ε=6.0)//X-D3(BJ)/LanL2DZ/6-31G* using different dispersion-parameterized DFT functionals X; the pre-S_0(W2-bound) and S_0^B(closed-cubane) states are set as the zero references for the two cases, respectively, in each functional.

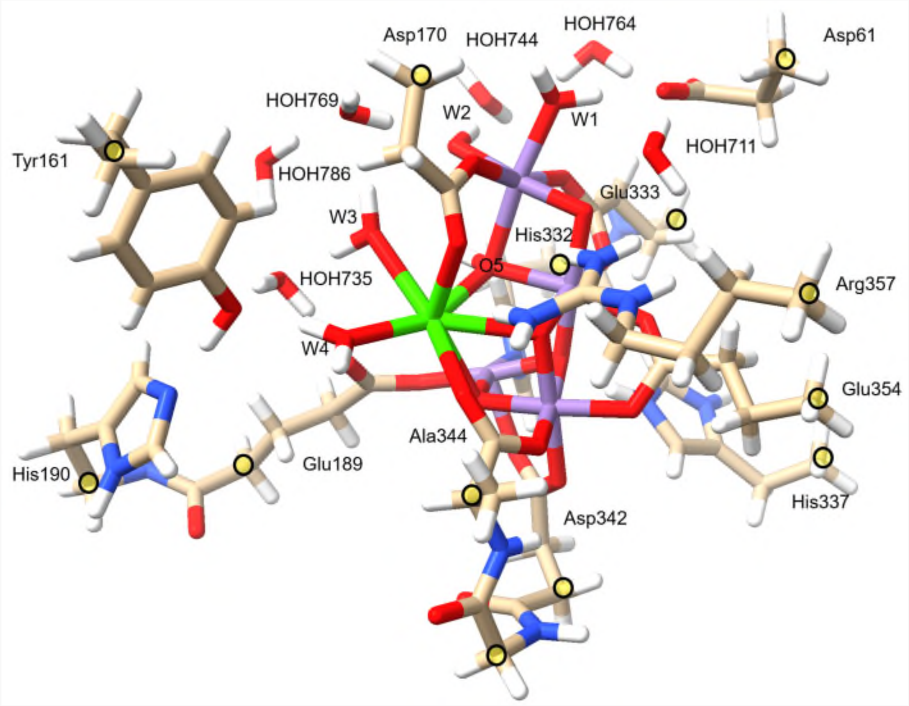

Suppl. Fig. 12. Model exhibition for MEP. The DFT model truncated from 6DHP including Asp61 for μ-O5H shift. The names of crystal waters are adjusted in 6DHP. The small yellow circles on the right denote the fixed α-carbon atoms.

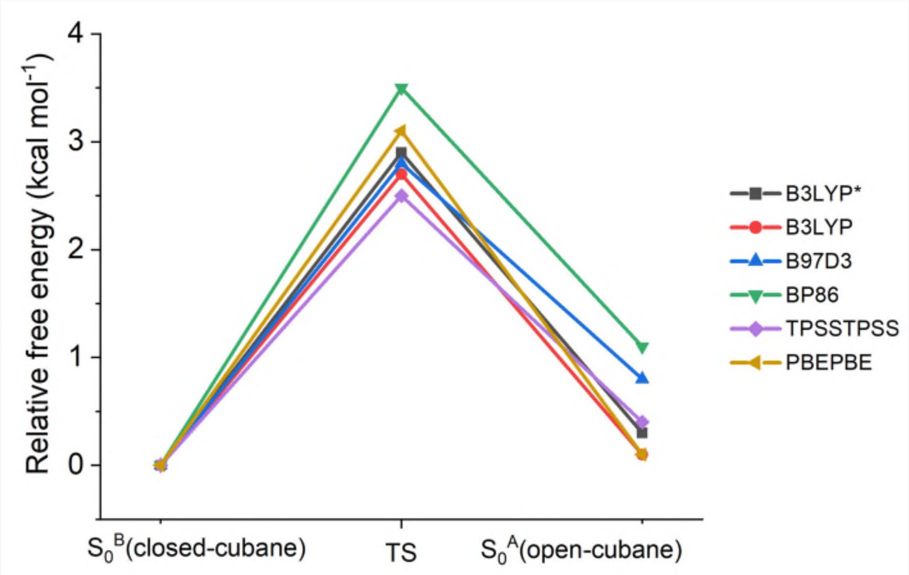

Suppl. Fig. 13. Functional sensitivity test. Functional dependence of the reaction energetics of the structural isomerism in the S_0 state by using the model including D1-Asp61 truncated from 6DHP, under the doublet/αβαβ spin state computed at the level of X-D3(BJ)/SDD/cc-pvtz(-*f*)/SMD(ε=6.0)//X-D3(BJ)/LanL2DZ/6-31G* using different dispersion-parameterized DFT functionals X; the pre-S_0(W2-bound) and S_0^B(closed-cubane) states are set as the zero references for the two cases, respectively, in each functional.

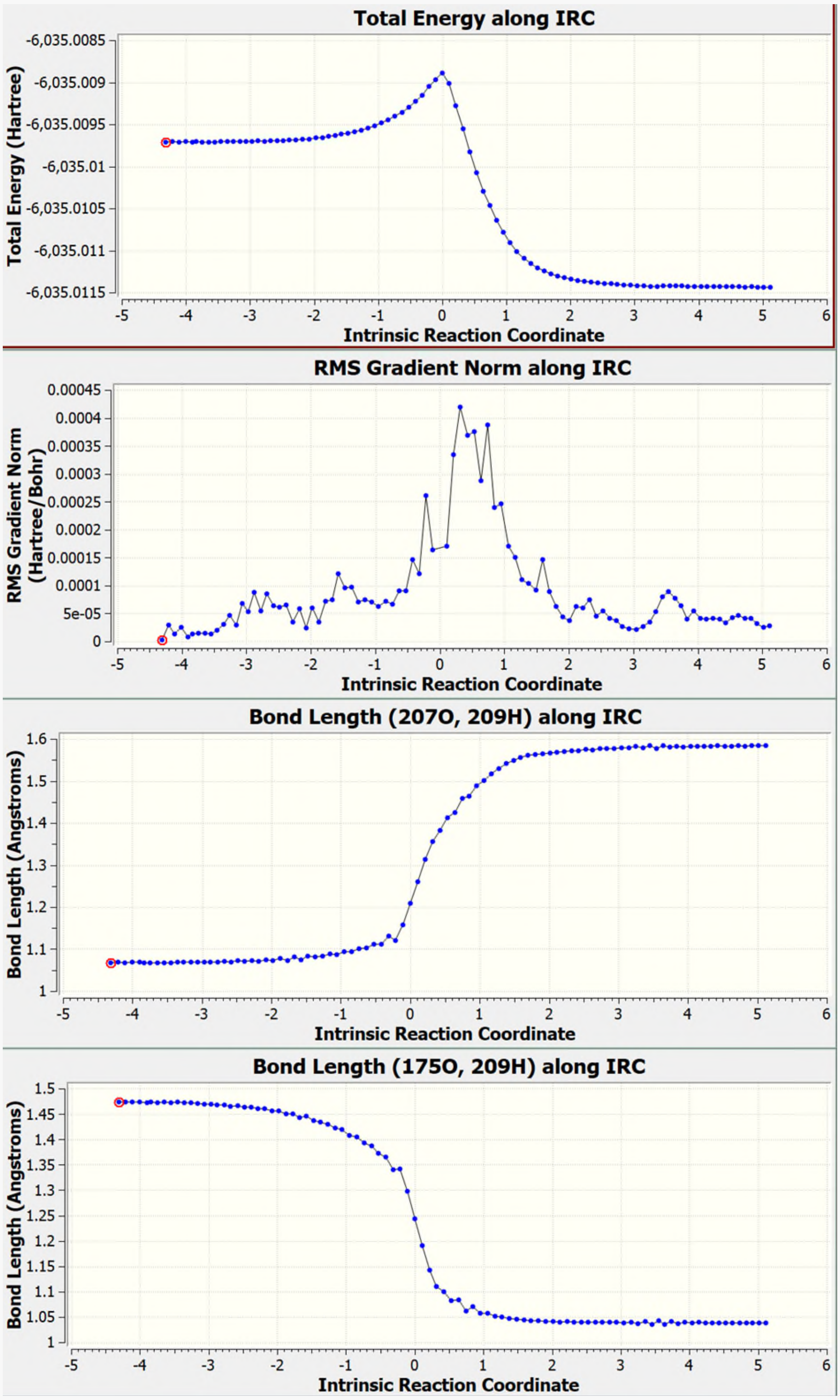

Suppl. Fig. 14. IRC. The IRC curve for W1 deprotonation to Asp61 for the octet/αααβ spin state with changes of the electronic energies (without ZPE and thermal corrections), RMS gradient norm and bond lengths of W1(207O)-H(209H) and Asp61(175O)-H(209H) along IRC.

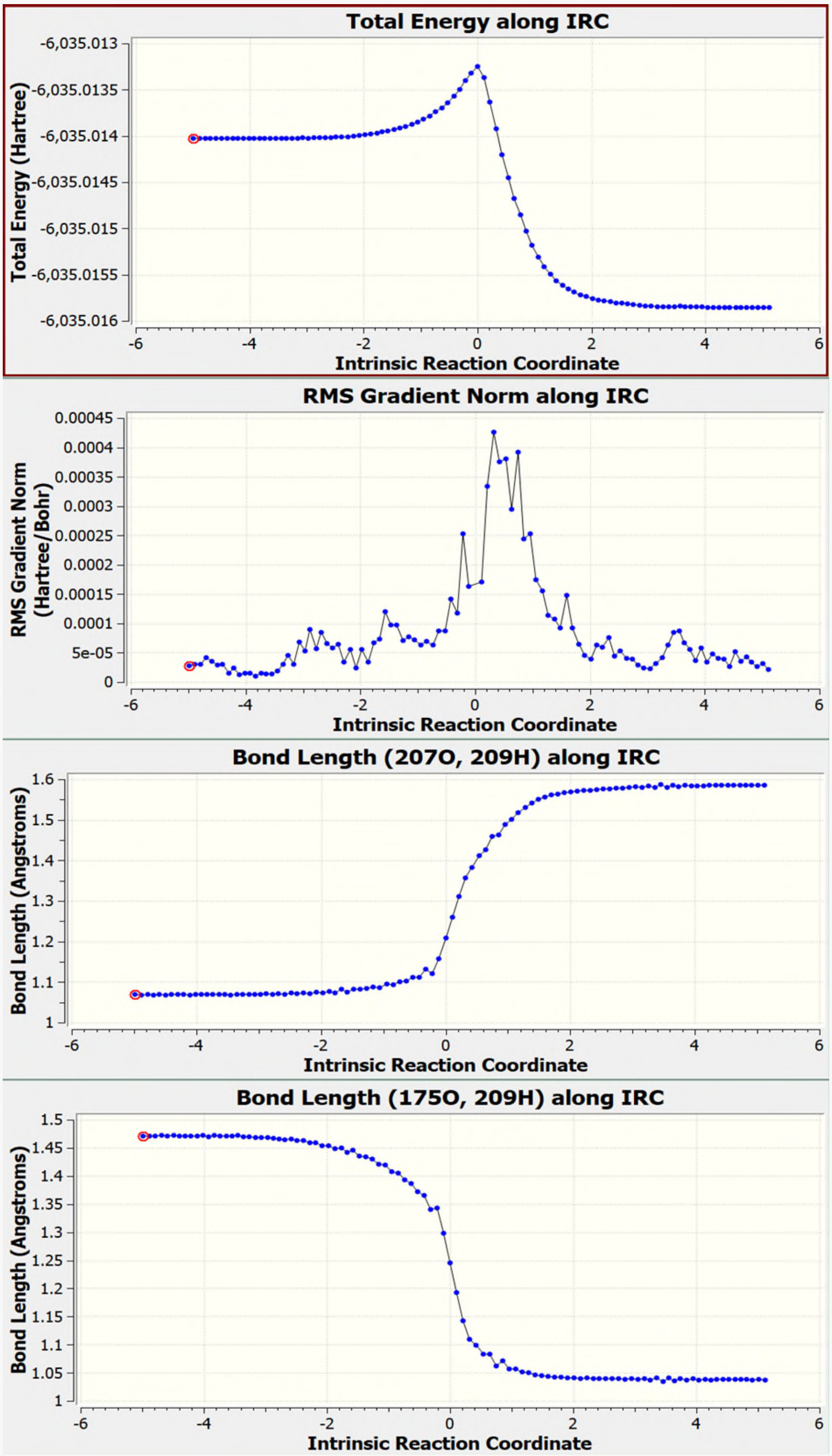

Suppl. Fig. 15. IRC. The IRC curve for W1 deprotonation to Asp61 for the doublet/αβαβ spin state with changes of the electronic energies (without ZPE and thermal corrections), RMS gradient norm and bond lengths of W1(207O)-H(209H) and Asp61(175O)-H(209H) along the IRC.

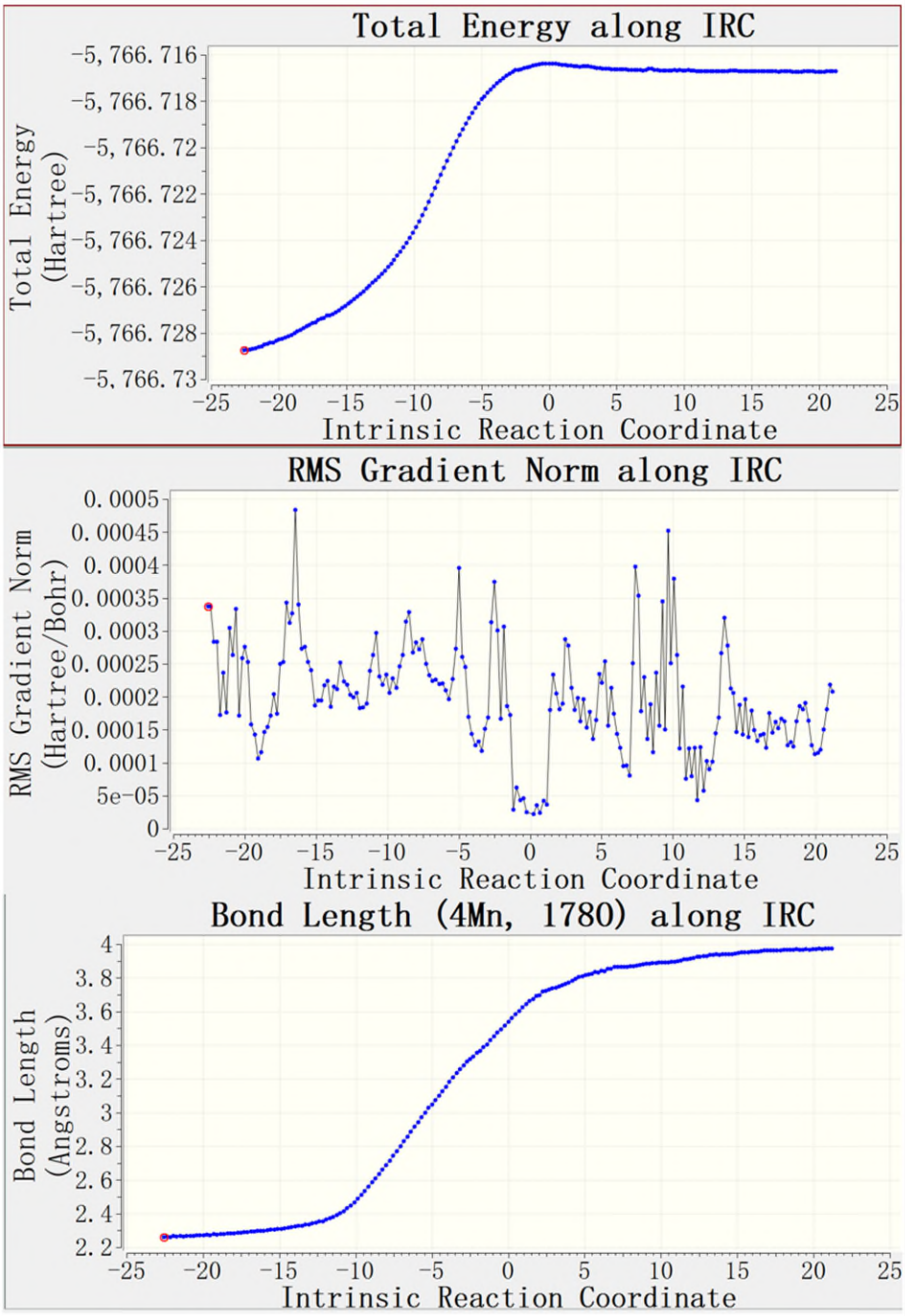

Suppl. Fig. 16. IRC. The IRC curve for W2 dissociation for the octet/αααβ spin state with changes of the electronic energies (without ZPE and thermal corrections), RMS gradient norm andbond lengths of Mn4-W2(178O) along the IRC.

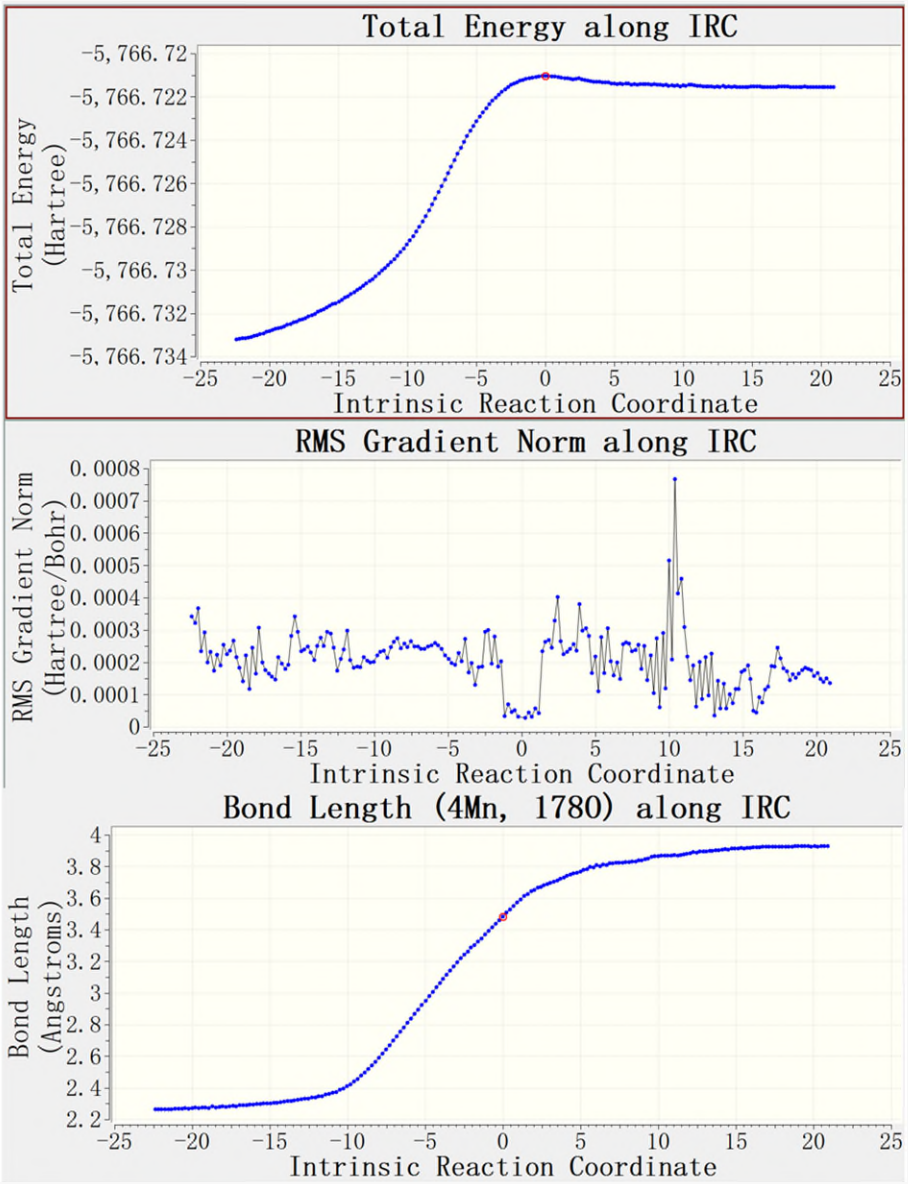

Suppl. Fig. 17. IRC. The IRC curve for W2 dissociation for the doublet/αβαβ spin state with changes of the electronic energies (without ZPE and thermal corrections), RMS gradient norm and bond lengths of Mn4-W2(178O) along the IRC.

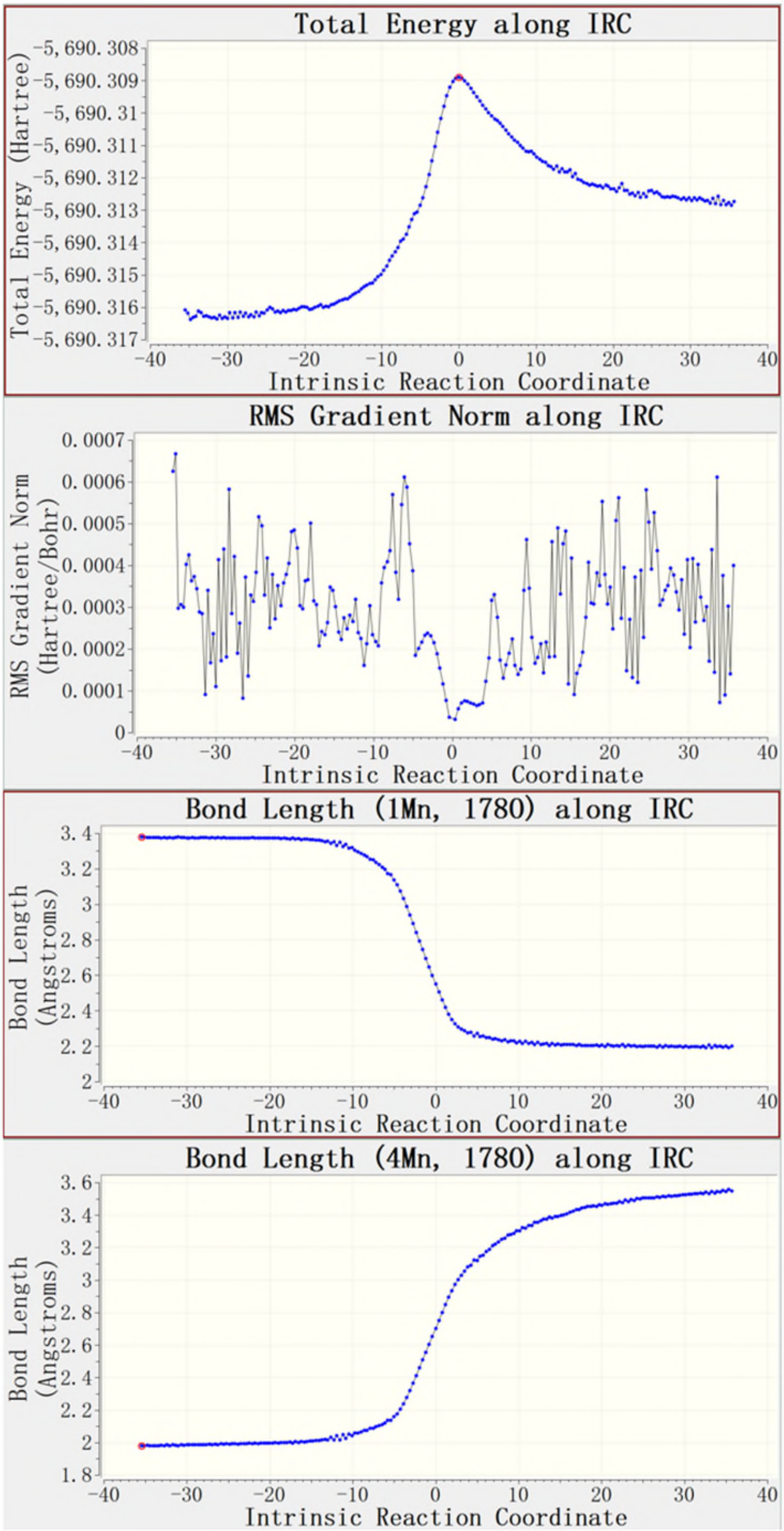

Suppl. Fig. 18. IRC. The IRC curve for the cluster isomerization in the S_0 state for the octet/αααβ

spin state with changes of the electronic energies (without ZPE and thermal corrections), RMS gradient norm and bond lengths of Mn1-W3(178O) and Mn4-W3(178O) along the IRC.

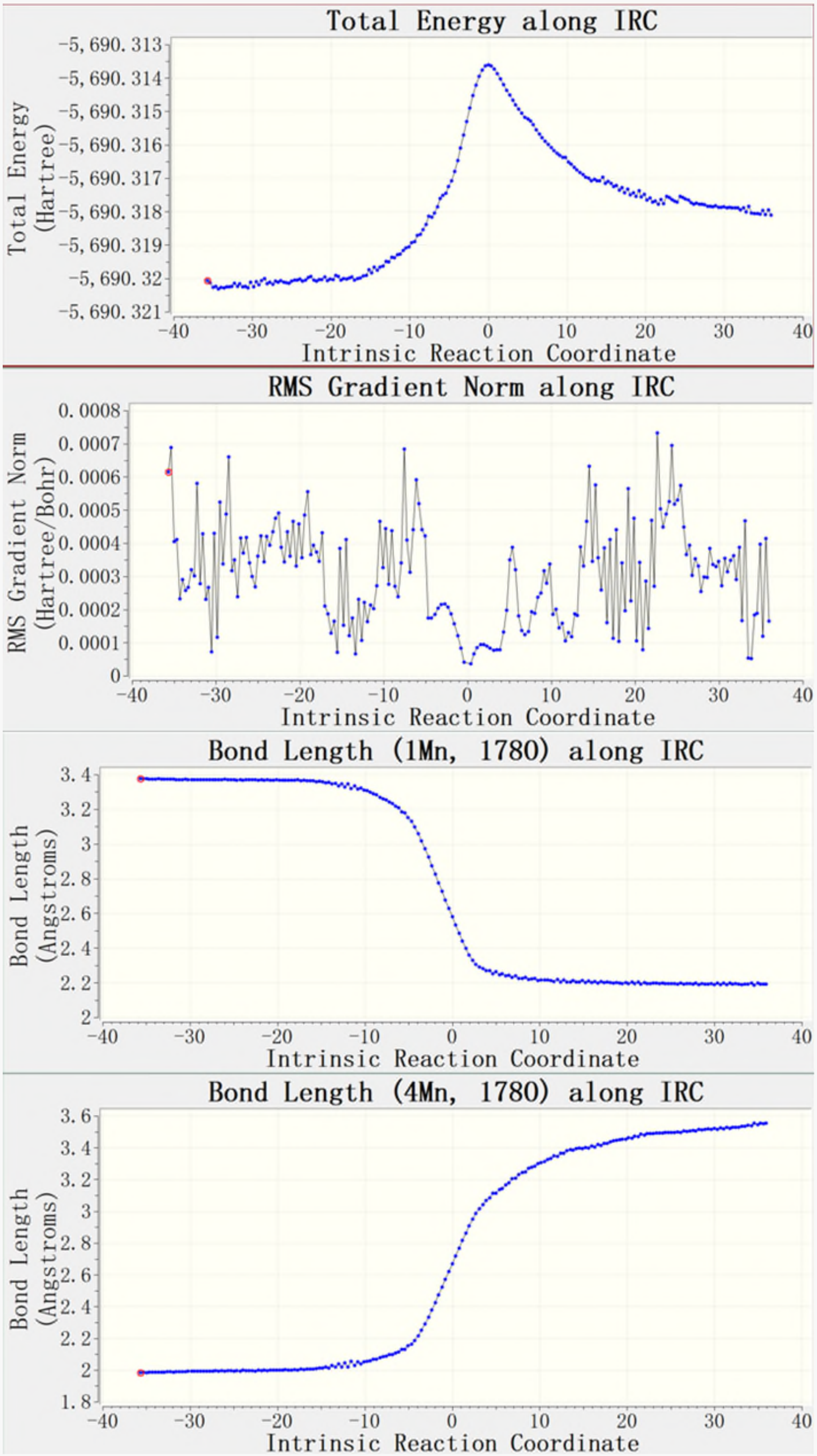

Suppl. Fig. 19. IRC. The IRC curve for the cluster isomerization in the S_0 state for the

doublet/αβαβ spin state with changes of the electronic energies (without ZPE and thermal corrections), RMS gradient norm and bond lengths of Mn1-W3(178O) and Mn4-W3(178O) along the IRC.

IV. The Fortran code used to collect the interatomic distances

```
program dist
implicit none
integer(8) :: nAtoms
integer(8) :: i,j,k,m,n
character(4), allocatable:: cElem(:)
real(8) :: rxv,x(100000),y(100000),z(100000)
real(8) :: dxv,dyv,dzv

allocate(cElem(900000))

open(unit=999, file='coors.xyz', status='old')
open(unit=998, file='W3-W5.txt', status='unknown')

do i=1,30000,1
read(999, *)
read(999, *)

do k = 1,369
     read(999,'(2x,a2,3x,3f16.10)') cElem(k),x(k),y(k),z(k)
enddo

do k= 1,369
      dxv=x(360)-x(303)
      dyv=y(360)-y(303)
      dzv=z(360)-z(303)
      rxv=sqrt(dxv*dxv+dyv*dyv+dzv*dzv)
 enddo

 write(998,'(1x,i5,3x,f6.4)') i, rxv

 enddo
 close(998)
 deallocate(cElem)
 end
```

V. Supplementary References

1. Bhowmick, A., *et al.* Structural evidence for intermediates during O_2 formation in photosystem II. *Nature* **617**, 629-636 (2023).
2. Greife, P., *et al.* The electron–proton bottleneck of photosynthetic oxygen evolution. *Nature* **617**, 623–628 (2023).
3. Suga, M., *et al.* An oxyl/oxo mechanism for oxygen-oxygen coupling in PSII revealed by an x-ray free-electron laser. *Science* **366**, 334-338 (2019).
4. de Lichtenberg, C., *et al.* Assignment of the slowly exchanging substrate water of nature's water-splitting cofactor. *Proc. Natl. Acad. Sci.* **121**, e2319374121 (2024).
5. de Lichtenberg, C., Kim, C.J., Chernev, P., Debus, R.J. & Messinger, J. The exchange of the fast substrate water in the S_2 state of photosystem II is limited by diffusion of bulk water through channels – implications for the water oxidation mechanism. *Chem. Sci.* **12**, 12763-12775 (2021).
6. Cox, N. & Messinger, J. Reflections on substrate water and dioxygen formation. *Biochim. Biophys. Acta, Bioenerg.* **1827**, 1020-1030 (2013).
7. Chernev, P., Aydin, A.O. & Messinger, J. On the simulation and interpretation of substrate-water exchange experiments in photosynthetic water oxidation. *Photosynth. Res.* (2024).
8. Siegbahn, P.E.M. Water oxidation mechanism in photosystem II, including oxidations, proton release pathways, O-O bond formation and O_2 release. *Biochim. Biophys. Acta, Bioenerg.* **1827**, 1003-1019 (2013).
9. Siegbahn, P.E.M. Structures and energetics for O_2 formation in photosystem II. *Acc. Chem. Res.* **42**, 1871-1880 (2009).
10. Guo, Y., *et al.* The open-cubane oxo-oxyl coupling mechanism dominates photosynthetic oxygen evolution: a comprehensive DFT investigation on O-O bond formation in the S_4 state. *Phys. Chem. Chem. Phys.* **19**, 13909-13923 (2017).
11. Rummel, F. & O'Malley, P.J. How nature makes O_2: an electronic level mechanism for water oxidation in photosynthesis. *J. Phys. Chem. B* **126**, 8214–8221 (2022).
12. Corry, T.A. & O'Malley, P.J. Electronic–level view of O–O bond formation in nature's water oxidizing complex. *J. Phys. Chem. Lett.* **11**, 4221-4225 (2020).
13. Shoji, M., Isobe, H., Shigeta, Y., Nakajima, T. & Yamaguchi, K. Nonadiabatic one-electron transfer mechanism for the O-O bond formation in the oxygen-evolving complex of photosystem II. *Chem. Phys. Lett.* **698**, 138-146 (2018).
14. Song, X. & Wang, B. O–O bond formation and oxygen release in photosystem II are enhanced by spin-exchange and synergetic coordination interactions. *J. Chem. Theory Comput.* **19**, 2684–2696 (2023).
15. Capone, M., Guidoni, L. & Narzi, D. Structural and dynamical characterization of the S_4 state of the Kok-Joliot's cycle by means of QM/MM molecular dynamics simulations. *Chem. Phys. Lett.* **742**, 137111 (2020).
16. Narzi, D., Capone, M., Bovi, D. & Guidoni, L. Evolution from S_3 to S_4 states of the oxygen-evolving complex in photosystem II monitored by quantum mechanics/molecular mechanics (QM/MM) dynamics. *Chem. Eur. J.* **24**, 10820-10828 (2018).
17. Allgöwer, F., Gamiz-Hernandez, A.P., Rutherford, A.W. & Kaila, V.R.I. Molecular

principles of redox-coupled protonation dynamics in photosystem II. *J. Am. Chem. Soc.* **144**, 7171–7180 (2022).
18. Simon, P.S., *et al.* Capturing the sequence of events during the water oxidation reaction in photosynthesis using XFELs. *FEBS Lett.* **597**, 30-37 (2023).
19. Guo, Y., Messinger, J., Kloo, L. & Sun, L. Alternative mechanism for O_2 formation in natural photosynthesis via nucleophilic oxo–oxo coupling. *J. Am. Chem. Soc.* **145**, 4129–4141 (2023).
20. Krewald, V., Neese, F. & Pantazis, D.A. Implications of structural heterogeneity for the electronic structure of the final oxygen-evolving intermediate in photosystem II. *J. Inorg. Biochem.* **199**, 110797 (2019).
21. Li, X. & Siegbahn, P.E.M. Alternative mechanisms for O_2 release and O-O bond formation in the oxygen evolving complex of photosystem II. *Phys. Chem. Chem. Phys.* **17**, 12168-12174 (2015).
22. Sproviero, E.M., Gascón, J.A., McEvoy, J.P., Brudvig, G.W. & Batista, V.S. Quantum mechanics/molecular mechanics study of the catalytic cycle of water splitting in photosystem II. *J. Am. Chem. Soc.* **130**, 3428-3442 (2008).
23. Kawashima, K., Takaoka, T., Kimura, H., Saito, K. & Ishikita, H. O_2 evolution and recovery of the water-oxidizing enzyme. *Nat. Commun.* **9**, 1247 (2018).
24. Zhang, B. & Sun, L. Why nature chose the Mn_4CaO_5 cluster as water-splitting catalyst in photosystem II: a new hypothesis for the mechanism of O-O bond formation. *Dalton Trans.* **47**, 14381-14387 (2018).
25. Shen, J.-R. The structure of photosystem II and the mechanism of water oxidation in photosynthesis. *Annu. Rev. Plant Biol.* **66**, 23-48 (2015).
26. Ames, W., *et al.* Theoretical evaluation of structural models of the S_2 state in the oxygen evolving complex of photosystem II: protonation states and magnetic interactions. *J. Am. Chem. Soc.* **133**, 19743-19757 (2011).
27. Krewald, V., *et al.* Metal oxidation states in biological water splitting. *Chem. Sci.* **6**, 1676-1695 (2015).
28. Robertazzi, A., Galstyan, A. & Knapp, E.W. PSII manganese cluster: protonation of W2, O5, O4 and His337 in the S_1 state explored by combined quantum chemical and electrostatic energy computations. *Biochim. Biophys. Acta, Bioenerg.* **1837**, 1316-1321 (2014).
29. Yang, K.R., Lakshmi, K.V., Brudvig, G.W. & Batista, V.S. Is deprotonation of the oxygen-evolving complex of photosystem II during the $S_1 \rightarrow S_2$ transition suppressed by proton quantum delocalization? *J. Am. Chem. Soc.* **143**, 8324-8332 (2021).
30. Isobe, H., *et al.* Theoretical illumination of water-inserted structures of the $CaMn_4O_5$ cluster in the S_2 and S_3 states of oxygen-evolving complex of photosystem II: full geometry optimizations by B3LYP hybrid density functional. *Dalton Trans.* **41**, 13727-13740 (2012).
31. Kusunoki, M. S_1-state Mn_4Ca complex of Photosystem II exists in equilibrium between the two most-stable isomeric substates: XRD and EXAFS evidence. *J. Photochem. Photobiol., B* **104**, 100-110 (2011).
32. Saito, K., Nakagawa, M. & Ishikita, H. pKa of the ligand water molecules in the oxygen-evolving Mn4CaO5 cluster in photosystem II. *Commun. Chem.* **3**, 89 (2020).

33. Siegbahn, P.E.M. Substrate water exchange for the oxygen evolving complex in PSII in the S_1, S_2, and S_3 states. *J. Am. Chem. Soc.* **135**, 9442-9449 (2013).
34. Isobe, H., Shoji, M., Suzuki, T., Shen, J.-R. & Yamaguchi, K. Spin, valence, and structural isomerism in the S_3 State of the oxygen-evolving complex of photosystem II as a manifestation of multimetallic cooperativity *J. Chem. Theory Comput.* **15**, 2375–2391 (2019).
35. Isobe, H., Shoji, M., Shen, J.-R. & Yamaguchi, K. Chemical equilibrium models for the S_3 state of the oxygen-evolving complex of photosystem II. *Inorg. Chem.* **55**, 502-511 (2016).
36. Zahariou, G., Ioannidis, N., Sanakis, Y. & Pantazis, D.A. Arrested substrate binding resolves catalytic intermediates in higher-plant water oxidation. *Angew. Chem. Int. Ed.* **60**, 3156-3162 (2021).
37. Drosou, M., Zahariou, G. & Pantazis, D.A. Orientational Jahn–Teller isomerism in the dark-stable state of nature’s water oxidase. *Angew. Chem. Int. Ed.* **60**, 13493-13499 (2021).
38. Retegan, M.*, et al.* A five-coordinate Mn(IV) intermediate in biological water oxidation: spectroscopic signature and a pivot mechanism for water binding. *Chem. Sci.* **7**, 72-84 (2016).
39. Krewald, V.*, et al.* Spin state as a marker for the structural evolution of nature's water-splitting catalyst. *Inorg. Chem.* **55**, 488-501 (2016).
40. Cox, N.*, et al.* Electronic structure of the oxygen-evolving complex in photosystem II prior to O-O bond formation. *Science* **345**, 804-808 (2014).
41. Shevela, D., Kern, J.F., Govindjee, G. & Messinger, J. Solar energy conversion by photosystem II: principles and structures. *Photosynth. Res.* **156**, 279–307 (2023).
42. Chrysina, M.*, et al.* Five-coordinate Mn^{IV} intermediate in the activation of nature’s water splitting cofactor. *Proc. Natl. Acad. Sci. U.S.A.* **116**, 16841-16846 (2019).
43. de Lichtenberg, C. & Messinger, J. Substrate water exchange in the S_2 state of photosystem II is dependent on the conformation of the Mn_4Ca cluster. *Phys. Chem. Chem. Phys.* **22**, 12894-12908 (2020).
44. Corry, T.A. & O’Malley, P.J. Proton isomers rationalize the high- and low-spin forms of the S_2 state intermediate in the water-oxidizing reaction of photosystem II. *J. Phys. Chem. Lett.* **10**, 5226-5230 (2019).
45. Guo, Y.*, et al.* How does ammonia bind to the oxygen-evolving complex in the S_2 state of photosynthetic water oxidation? Theoretical support and implications for the W1 substitution mechanism. *Phys. Chem. Chem. Phys.* **18**, 31551-31565 (2016).
46. Pushkar, Y., Ravari, A.K., Jensen, S.C. & Palenik, M. Early binding of substrate oxygen is responsible for a spectroscopically distinct S_2 state in photosystem II. *J. Phys. Chem. Lett.* **10**, 5284-5291 (2019).
47. Capone, M., Narzi, D. & Guidoni, L. Mechanism of oxygen evolution and Mn_4CaO_5 cluster restoration in the natural water-oxidizing catalyst. *Biochemistry* **60**, 2341–2348 (2021).
48. Capone, M., Bovi, D., Narzi, D. & Guidoni, L. Reorganization of substrate waters between the closed and open cubane conformers during the S_2 to S_3 transition in the oxygen evolving complex. *Biochemistry* **54**, 6439-6442 (2015).

49. Narzi, D., Bovi, D. & Guidoni, L. Pathway for Mn-cluster oxidation by tyrosine-Z in the S_2 state of photosystem II. *Proc. Natl. Acad. Sci. U.S.A.* **111**, 8723-8728 (2014).
50. Bovi, D., Narzi, D. & Guidoni, L. The S_2 state of the oxygen-evolving complex of photosystem II explored by QM/MM dynamics: spin surfaces and metastable states suggest a reaction path towards the S_3 state. *Angew. Chem. Int. Ed.* **52**, 1-6 (2013).
51. Yamamoto, M., Nakamura, S. & Noguchi, T. Protonation structure of the photosynthetic water oxidizing complex in the S_0 state as revealed by normal mode analysis using quantum mechanics/molecular mechanics calculations. *Phys. Chem. Chem. Phys.* **22**, 24213-24225 (2020).
52. Nakamura, S. & Noguchi, T. Quantum mechanics/molecular mechanics simulation of the ligand vibrations of the water-oxidizing Mn_4CaO_5 cluster in photosystem II. *Proc. Natl. Acad. Sci. U.S.A.* **113**, 12727-12732 (2016).
53. Chrysina, M.*, et al.* Nature of S-states in the oxygen-evolving complex resolved by high-energy resolution fluorescence detected X-ray absorption spectroscopy. *J. Am. Chem. Soc.* **145**, 25579–25594 (2023).
54. Yamaguchi, K.*, et al.* Theoretical elucidation of the structure, bonding, and reactivity of the $CaMn_4Ox$ clusters in the whole Kok cycle for water oxidation embedded in the oxygen evolving center of photosystem II. New molecular and quantum insights into the mechanism of the O–O bond formation. *Photosynth. Res.* (2023).
55. Yamaguchi, K.*, et al.* Geometric, electronic and spin structures of the $CaMn_4O_5$ catalyst for water oxidation in oxygen-evolving photosystem II. Interplay between experiments and theoretical computations. *Coord. Chem. Rev.* **471**, 214742 (2022).
56. Rummel, F., Malcomson, T., Barchenko, M. & O'Malley, P.J. Insights into PSII's $S_3Y_Z^{\bullet}$ state: an electronic and magnetic analysis. *J. Phys. Chem. Lett.* **15**, 499-506 (2024).
57. Saito, K., Nakao, S. & Ishikita, H. Identification of the protonation and oxidation states of the oxygen-evolving complex in the low-dose X-ray crystal structure of photosystem II. *Front. Plant Sci.* **14**(2023).
58. Isobe, H.*, et al.* Generalized approximate spin projection calculations of effective exchange integrals of the $CaMn_4O_5$ cluster in the S_1 and S_3 states of the oxygen evolving complex of photosystem II. *Phys. Chem. Chem. Phys.* **16**, 11911-11923 (2014).
59. Siegbahn, P.E.M. Water oxidation in photosystem II: oxygen release, proton release and the effect of chloride. *Dalton Trans.*, 10063-10068 (2009).
60. Hung, S.-W., Yang, F.-A., Chen, J.-H., Wang, S.-S. & Tung, J.-Y. Magnetic susceptibility and ground-state zero-field splitting in high-spin mononuclear manganese(III) of inverted N-methylated porphyrin complexes: Mn(2-NCH_3NCTPP)Br_$^{\#}$. *Inorg. Chem.* **47**, 7202-7206 (2008).
61. Darensbourg, D.J. & Frantz, E.B. Manganese(III) schiff base complexes: chemistry relevant to the copolymerization of epoxides and carbon dioxide. *Inorg. Chem.* **46**, 5967-5978 (2007).
62. Bellemin-Laponnaz, S. & Dagorne, S. Group 1 and 2 and early transition metal complexes bearing N-heterocyclic carbene ligands: coordination chemistry, reactivity, and applications. *Chem. Rev.* **114**, 8747-8774 (2014).
63. Shoji, M., Isobe, H., Shigeta, Y., Nakajima, T. & Yamaguchi, K. Concerted mechanism of water insertion and O_2 release during the S_4 to S_0 transition of the oxygen-evolving

complex in photosystem II. *J. Phys. Chem. B* **122**, 6491-6502 (2018).
64. Messinger, J.*, et al.* The S_0 state of the oxygen-evolving complex in photosystem II Is paramagnetic: detection of an EPR multiline signal. *J. Am. Chem. Soc.* **119**, 11349-11350 (1997).
65. Messinger, J., Nugent, J.H.A. & Evans, M.C.W. Detection of an EPR multiline signal for the S_0* state in photosystem II. *Biochemistry* **36**, 11055-11060 (1997).
66. Åhrling, K.A., Peterson, S. & Styring, S. An oscillating manganese electron paramagnetic resonance signal from the S_0 state of the oxygen evolving complex in photosystem II. *Biochemistry* **36**, 13148-13152 (1997).
67. Boussac, A., Kuhl, H., Ghibaudi, E., Rögner, M. & Rutherford, A.W. Detection of an electron paramagnetic resonance signal in the S_0 state of the manganese complex of photosystem II from Synechococcus elongatus. *Biochemistry* **38**, 11942-11948 (1999).
68. Robblee, J.H.*, et al.* The Mn cluster in the S_0 state of the oxygen-evolving complex of photosystem II studied by EXAFS spectroscopy: are there three di-μ-oxo-bridged Mn_2 moieties in the tetranuclear Mn complex? *J. Am. Chem. Soc.* **124**, 7459-7471 (2002).
69. Kulik, L.V., Epel, B., Lubitz, W. & Messinger, J. ^{55}Mn Pulse ENDOR at 34 GHz of the S_0 and S_2 States of the oxygen-evolving complex in photosystem II. *J. Am. Chem. Soc.* **127**, 2392-2393 (2005).
70. Kulik, L.V., Epel, B., Lubitz, W. & Messinger, J. Electronic structure of the Mn_4OxCa cluster in the S_0 and S_2 states of the oxygen-evolving complex of photosystem II based on pulse ^{55}Mn-ENDOR and EPR spectroscopy. *J. Am. Chem. Soc.* **129**, 13421-13435 (2007).
71. Lohmiller, T.*, et al.* The first state in the catalytic cycle of the water-oxidizing enzyme: identification of a water-derived μ-hydroxo bridge. *J. Am. Chem. Soc.* **139**, 14412-14424 (2017).
72. Cheah, M.H.*, et al.* Assessment of the manganese cluster's oxidation state via photoactivation of photosystem II microcrystals. *Proc. Natl. Acad. Sci. U.S.A.* **117**, 141-145 (2020).
73. Askerka, M., Wang, J., Vinyard, D.J., Brudvig, G.W. & Batista, V.S. S_3 state of the O_2-evolving complex of photosystem II: insights from QM/MM, EXAFS, and femtosecond X-ray diffraction. *Biochemistry* **55**, 981-984 (2016).
74. Askerka, M., Vinyard, D.J., Brudvig, G.W. & Batista, V.S. NH_3 binding to the S_2 state of the O_2-evolving complex of photosystem II: analogue to H_2O binding during the $S_2 \rightarrow S_3$ transition. *Biochemistry* **54**, 5783-5786 (2015).
75. Wang, J., Askerka, M., Brudvig, G.W. & Batista, V.S. Crystallographic data support the carousel mechanism of water supply to the oxygen-evolving complex of photosystem II. *ACS Energy Lett.* **2**, 2299–2306 (2017).
76. Capone, M., Narzi, D., Bovi, D. & Guidoni, L. Mechanism of water delivery to the active site of photosystem II along the S_2 to S_3 transition. *J. Phys. Chem. Lett.* **7**, 592-596 (2016).
77. Quagliano, J.V. & Schubert, L.E.O. The trans effect in complex inorganic compounds. *Chem. Rev.* **50**, 201-260 (1952).
78. Shustorovich, E.M., Porai-Koshits, M.A. & Buslaev, Y.A. The mutual influence of ligands in transition metal coordination compounds with multiple metal-ligand bonds. *Coord. Chem. Rev.* **17**, 1-98 (1975).
79. Burdett, J.K. & Albright, T.A. Trans influence and mutual influence of ligands

coordinated to a central atom. *Inorg. Chem.* **18**, 2112-2120 (1979).
80. Coe, B.J. & Glenwright, S.J. Trans-effects in octahedral transition metal complexes. *Coord. Chem. Rev.* **203**, 5-80 (2000).
81. Rivalta, I., *et al.* Structural-functional role of chloride in photosystem II. *Biochemistry* **50**, 6312-6315 (2011).
82. Ahmadi, S., *et al.* Multiscale modeling of enzymes: QM-cluster, QM/MM, and QM/MM/MD: A tutorial review. *Int J Quantum Chem.* **118**, e25558 (2018).
83. Blomberg, M.R.A., Borowski, T., Himo, F., Liao, R.-Z. & Siegbahn, P.E.M. Quantum chemical studies of mechanisms for metalloenzymes. *Chem. Rev.* **114**, 3601-3658 (2014).
84. Saito, K., Rutherford, A.W. & Ishikita, H. Energetics of proton release on the first oxidation step in the water-oxidizing enzyme. *Nat. Commun.* **6**, 8488 (2015).
85. Shoji, M., Isobe, H. & Yamaguchi, K. QM/MM study of the S_2 to S_3 transition reaction in the oxygen-evolving complex of photosystem II. *Chem. Phys. Lett.* **636**, 172-179 (2015).
86. Sproviero, E.M., *et al.* QM/MM computational studies of substrate water binding to the oxygen-evolving centre of photosystem II. *Philos. Trans. R. Soc., B* **363**, 1149-1156 (2008).
87. Kim, C.J. & Debus, R.J. Evidence from FTIR difference spectroscopy that a substrate H_2O molecule for O_2 formation in photosystem II is provided by the Ca ion of the catalytic Mn_4CaO_5 cluster. *Biochemistry* **56**, 2558-2570 (2017).
88. Kim, C.J. & Debus, R.J. One of the substrate waters for O_2 formation in photosystem II is provided by the water-splitting Mn_4CaO_5 cluster's Ca^{2+} ion. *Biochemistry* **58**, 3185-3192 (2019).
89. Zaharieva, I. & Dau, H. Energetics and kinetics of S-State transitions monitored by delayed chlorophyll fluorescence. *Front. Plant Sci.* **10**, 386 (2019).
90. Klauss, A., Haumann, M. & Dau, H. Seven steps of alternating electron and proton transfer in photosystem II water oxidation traced by time-resolved photothermal beam deflection at improved sensitivity. *J. Phys. Chem. B* **119**, 2677-2689 (2015).
91. Nilsson, H., Cournac, L., Rappaport, F., Messinger, J. & Lavergne, J. Estimation of the driving force for dioxygen formation in photosynthesis. *Biochim. Biophys. Acta, Bioenerg.* **1857**, 23-33 (2016).
92. Marcus, Y. Thermodynamic functions of transfer of single ions from water to nonaqueous and mixed solvents: Part 2 - Enthalpies and entropies of transfer to nonaqueous solvents. *Pure Appl. Chem.* **57**, 1103-1128 (1985).
93. Debus, R.J. Evidence from FTIR difference spectroscopy that D1-Asp61 influences the water reactions of the oxygen-evolving Mn_4CaO_5 cluster of photosystem II. *Biochemistry* **53**, 2941-2955 (2014).
94. Hussein, R., *et al.* Structural dynamics in the water and proton channels of photosystem II during the S_2 to S_3 transition. *Nat. Commun.* **12**, 6531 (2021).
95. Shimada, Y., Sugiyama, A., Nagao, R. & Noguchi, T. Role of D1-Glu65 in Proton Transfer during Photosynthetic Water Oxidation in Photosystem II. *J. Phys. Chem. B* (2022).
96. Siegbahn, P.E.M. The performance of hybrid DFT for mechanisms involving transition metal complexes in enzymes. *J. Biol. Inorg. Chem.* **11**, 695–701 (2006).
97. Siegbahn, P.E.M. & Blomberg, M.R.A. Transition-metal systems in biochemistry studied

by high-accuracy quantum chemical methods. *Chem. Rev.* **100**, 421-437 (2000).
98. Cramer, C.J. & Truhlar, D.G. Density functional theory for transition metals and transition metal chemistry. *Phys. Chem. Chem. Phys.* **11**, 10757-10816 (2009).
99. Claeyssens, F., *et al.* High-accuracy computation of reaction barriers in enzymes. *Angew. Chem. Int. Ed.* **45**, 6856 -6859 (2006).
100. Harvey, J.N., Poli, R. & Smith, K.M. Understanding the reactivity of transition metal complexes involving multiple spin states. *Coord. Chem. Rev.* **238-239**, 347-361 (2003).
101. Neese, F. Prediction of molecular properties and molecular spectroscopy with density functional theory: From fundamental theory to exchange-coupling. *Coord. Chem. Rev.* **253**, 526-563 (2009).
102. Cheong, P.H.-Y., Legault, C.Y., Um, J.M., Çelebi-Ölçüm, N. & Houk, K.N. Quantum mechanical investigations of organocatalysis: mechanisms, reactivities, and selectivities. *Chem. Rev.* **111**, 5042-5137 (2011).
103. Cheng, G.-J., Zhang, X., Chung, L.W., Xu, L. & Wu, Y.-D. Computational organic chemistry: bridging theory and experiment in establishing the mechanisms of chemical reactions. *J. Am. Chem. Soc.* **137**, 1706-1725 (2015).
104. Retegan, M., Neese, F. & Pantazis, D.A. Convergence of QM/MM and cluster models for the spectroscopic properties of the oxygen-evolving complex in photosystem II. *J. Chem. Theory Comput.* **9**, 3832-3842 (2013).
105. Schwiedrzik, L., Rajkovic, T. & González, L. Regeneration and degradation in a biomimetic polyoxometalate water oxidation catalyst. *ACS Catal.* **13**, 3007-3019 (2023).
106. Siegbahn, P.E.M. & Blomberg, M.R.A. Energy diagrams for water oxidation in photosystem II using different density functionals. *J. Chem. Theory Comput.* **10**, 268-272 (2014).
107. Siegbahn, P.E.M., Blomberg, M.R.A. & Chen, S.-L. Significant van der waals effects in transition metal complexes. *J. Chem. Theory Comput.* **6**, 2040-2044 (2010).
108. Grimme, S., Ehrlich, S. & Goerigk, L. Effect of the damping function in dispersion corrected density functional theory. *J. Comput. Chem.* **32**, 1456-1465 (2011).
109. Schwabe, T. & Grimme, S. Double-hybrid density functionals with long-range dispersion corrections: higher accuracy and extended applicability. *Phys. Chem. Chem. Phys.* **9**, 3397-3406 (2007).
110. Grimme, S. Semiempirical GGA-type density functional constructed with a long-range dispersion correction. *J. Comp. Chem.* **27**, 1787-1799 (2006).
111. Siegbahn, P.E.M. The S_2 to S_3 transition for water oxidation in PSII (photosystem II), revisited. *Phys. Chem. Chem. Phys.* **20**, 22926-22931 (2018).
112. Siegbahn, P.E.M. Nucleophilic water attack is not a possible mechanism for O-O bond formation in photosystem II. *Proc. Natl. Acad. Sci. U.S.A.* **114**, 4966-4968 (2017).
113. Siegbahn, P.E.M. O-O bond formation in the S_4 state of the oxygen-evolving complex in photosystem II. *Chem. Eur. J.* **12**, 9217-9227 (2006).
114. Li, M. & Liao, R.-Z. Water oxidation catalyzed by a bioinspired tetranuclear manganese complex: mechanistic study and prediction. *ChemSusChem* **15**, e202200187 (2022).
115. Zhang, H.-T., Su, X.-J., Xie, F., Liao, R.-Z. & Zhang, M.-T. Iron-catalyzed water oxidation: O–O Bond formation via intramolecular oxo–oxo interaction. *Angew. Chem. Int. Ed.* **60**, 12467-12474 (2021).

116. Chen, Q.-F., Cheng, Z.-Y., Liao, R.-Z. & Zhang, M.-T. Bioinspired trinuclear copper catalyst for water oxidation with a turnover frequency up to 20000 s^{-1}. *J. Am. Chem. Soc.* **143**, 19761-19768 (2021).
117. Liao, R.-Z. & Siegbahn, P.E.M. Possible water association and oxidation mechanisms for a recently synthesized Mn4Ca-complex. *J. Catal.* **354** 169-181 (2017).
118. Liao, R.-Z., Kärkäs, M.D., Lee, B.-L., Åkermark, B. & Siegbahn, P.E.M. Photosystem II like water oxidation mechanism in a bioinspired tetranuclear manganese complex. *Inorg. Chem.* **54**, 342-351 (2015).
119. Yeo, C., Nguyen, M. & Wang, L.-P. Benchmarking density functionals, basis sets, and solvent models in predicting thermodynamic organic hydrides. *J. Phys. Chem. A* **126**, 7566-7577 (2022).
120. Kern, J*., et al.* Structures of the intermediates of Kok's photosynthetic water oxidation clock. *Nature* **563**, 421-425 (2018).
121. Siegbahn, P.E.M. & Himo, F. The quantum chemical cluster approach for modeling enzyme reactions. *WIREs Comput. Mol. Sci.* **1**, 323-336 (2011).
122. Siegbahn, P.E.M. & Blomberg, M.R.A. Density functional theory of biologically relevant metal centers. *Annu. Rev. Phys. Chem.* **50**, 221-249 (1999).
123. Cohen, A.J., Mori-Sánchez, P. & Yang, W. Challenges for density functional theory. *Chem. Rev.* **112**, 289-320 (2012).
124. Ghosh, A. Just how good is DFT? *J. Biol. Inorg. Chem.* **11**, 671-673 (2006).
125. Pantazis, D.A., Ames, W., Cox, N., Lubitz, W. & Neese, F. Two interconvertible structures that explain the spectroscopic properties of the oxygen-evolving complex of photosystem II in the S_2 state. *Angew. Chem. Int. Ed.* **51**, 9935-9940 (2012).
126. Ugur, I., Rutherford, A.W. & Kaila, V.R.I. Redox-coupled substrate water reorganization in the active site of photosystem II-the role of calcium in substrate water delivery. *Biochim. Biophys. Acta, Bioenerg.* **1857**, 740-748 (2016).
127. Boussac, A*., et al.* The low spin-high spin equilibrium in the S_2-state of the water oxidizing enzyme. *Biochim. Biophys. Acta Bioenerg.* **1859**, 342-356 (2018).
128. Vinyard, D.J., Khan, S., Askerka, M., Batista, V.S. & Brudvig, G.W. Energetics of the S_2 state spin isomers of the oxygen-evolving complex of photosystem II. *J. Phys. Chem. B* **121**, 1020-1025 (2017).
129. Saitow, M., Becker, U., Riplinger, C., Valeev, E.F. & Neese, F. A new near-linear scaling, efficient and accurate, open-shell domain-based local pair natural orbital coupled cluster singles and doubles theory. *J. Chem. Phys.* **146**, 164105 (2017).
130. Isobe, H., Shoji, M., Suzuki, T., Shen, J.-R. & Yamaguchi, K. Exploring reaction pathways for the structural rearrangements of the Mn cluster induced by water binding in the S3 state of the oxygen evolving complex of photosystem II. *J. Photochem. Photobiol., A* **405**, 112905 (2021).
131. Guo, Y., Messinger, J., Kloo, L. & Sun, L. Reversible structural isomerization of nature's water oxidation catalyst prior to O–O bond formation. *J. Am. Chem. Soc.* **144**, 11736–11747 (2022).
132. Nilsson, H., Krupnik, T., Kargul, J. & Messinger, J. Substrate water exchange in photosystem II core complexes of the extremophilic red alga cyanidioschyzon merolae. *Biochim. Biophys. Acta, Bioenerg.* **1837**, 1257-1262 (2014).

133. de Lichtenberg, C., *et al.* The D1-V185N mutation alters substrate water exchange by stabilizing alternative structures of the Mn4Ca-cluster in photosystem II. *Biochim. Biophys. Acta, Bioenerg.* **1862**, 148319 (2021).
134. Taguchi, S., Noguchi, T. & Mino, H. Molecular structure of the S_2 state with a g = 5 signal in the oxygen evolving complex of photosystem II. *J. Phys. Chem. B* **124**, 5531-5537 (2020).
135. Mino, H. & Nagashima, H. Orientation of ligand field for dangling manganese in photosynthetic oxygen-evolving complex of photosystem II. *J. Phys. Chem. B* **124**, 128-133 (2020).
136. Boussac, A., Girerd, J.-J. & Rutherford, A.W. Conversion of the spin state of the manganese complex in photosystem II induced by near-infrared light. *Biochemistry* **35**, 6984-6989 (1996).
137. Boussac, A., Kuhl, H., Un, S., Rögner, M. & Rutherford, A.W. Effect of near-infrared light on the S_2-state of the manganese complex of photosystem II from Synechococcus elongatus. *Biochemistry* **37**, 8995-9000 (1998).
138. Boussac, A., Un, S., Horner, O. & Rutherford, A.W. High-spin states ($S \geq 5/2$) of the photosystem II manganese complex. *Biochemistry* **37**, 4001-4007 (1998).
139. Isobe, H., Shoji, M., Shen, J.-R. & Yamaguchi, K. Strong coupling between the hydrogen bonding environment and redox chemistry during the S_2 to S_3 transition in the oxygen-evolving complex of photosystem II. *J. Phys. Chem. B* **119**, 13922-13933 (2015).
140. Guo, Y., *et al.* Theoretical reflections on the structural polymorphism of the oxygen-evolving complex in the S_2 state and the correlations to substrate water exchange and water oxidation mechanism in photosynthesis. *Biochim. Biophys. Acta, Bioenerg.* **1858**, 833-846 (2017).
141. Saito, K., Mino, H., Nishio, S. & Ishikita, H. Protonation structure of the closed-cubane conformation of the O_2-evolving complex in photosystem II. *PNAS Nexus* **1**, 1-14 (2022).
142. Kosaki, S. & Mino, H. Molecular structure related to an S = 5/2 high-spin S_2 state manganese cluster of photosystem II investigated by Q-band pulse EPR spectroscopy. *J. Phys. Chem. B* **127**, 6441-6448 (2023).
143. Ibrahim, M., *et al.* Untangling the sequence of events during the $S_2 \rightarrow S_3$ transition in photosystem II and implications for the water oxidation mechanism. *Proc. Natl. Acad. Sci. U.S.A.* **117**, 12624-12635 (2020).
144. Chatterjee, R., *et al.* Structural isomers of the S_2 state in photosystem II: do they exist at room temperature and are they important for function? *Physiol. Plant.* **166**, 60-72 (2019).
145. Li, H., *et al.* Capturing structural changes of the S_1 to S_2 transition of photosystem II using time-resolved serial femtosecond crystallography. *IUCrJ* **8**, 431-443 (2021).
146. Suga, M., *et al.* Light-induced structural changes and the site of O=O bond formation in PSII caught by XFEL. *Nature* **543**, 131-135 (2017).
147. Suga, M., *et al.* Native structure of photosystem II at 1.95Å resolution viewed by femtosecond X-ray pulses. *Nature* **517**, 99-103 (2015).
148. Cox, N., Pantazis, D.A. & Lubitz, W. Current understanding of the mechanism of water oxidation in photosystem II and its relation to XFEL data. *Annu. Rev. Biochem.* **89**, 19.11-19.26 (2020).
149. Nakamura, S., Ota, K., Shibuya, Y. & Noguchi, T. Role of a water network around the

Mn_4CaO_5 cluster in photosynthetic water oxidation: a Fourier transform infrared spectroscopy and quantum mechanics/molecular mechanics calculation study. *Biochemistry* **55**, 597-607 (2016).

150. Fukui, K. The path of chemical-reactions-The IRC approach. *Acc. Chem. Res.* **14**, 363-368 (1981).

151. Schlegel, H.B. Optimization of equilibrium geometries and transition structures. *J. Comp. Chem.* **3**, 214-218 (1982).

152. Peng, C., Ayala, P.Y., Schlegel, H.B. & Frisch, M.J. Using redundant internal coordinates to optimize equilibrium geometries and transition states. *J. Comp. Chem.* **17**, 49-56 (1996).

153. Hratchian, H.P. & Schlegel, H.B. Accurate reaction paths using a Hessian based predictor–corrector integrator. *J. Chem. Phys.* **120**, 9918-9924 (2004).

154. Simons, J., Joergensen, P., Taylor, H. & Ozment, J. Walking on potential energy surfaces. *J. Phys. Chem.* **87**, 2745-2753 (1983).

155. Henkelman, G., Uberuaga, B.P. & Jónsson, H. A climbing image nudged elastic band method for finding saddle points and minimum energy paths. *J. Phys. Chem.* **113**, 9901-9904 (2000).

156. Guerra, F., Siemers, M., Mielack, C. & Bondar, A.-N. Dynamics of long-distance hydrogen-bond networks in photosystem II. *J. Phys. Chem. B* **122**, 4625-4641 (2018).

157. Feyel, S., *et al.* Activation of methane by oligomeric $(Al_2O_3)_x^+$ (x=3,4,5): the role of oxygen-centered radicals in thermal hydrogen-atom abstraction. *Angew. Chem. Int. Ed.* **47**, 1946-1950 (2008).

158. Wu, X.-N., *et al.* Active sites of stoichiometric cerium oxide cations ($Ce_mO_{2m}^+$) probed by reactions with carbon monoxide and small hydrocarbon molecules. *Phys. Chem. Chem. Phys.* **12**, 3984-3997 (2010).

159. Zhao, Y.-X., Wu, X.-N., Ma, J.-B., He, S.-G. & Ding, X.-L. Experimental and theoretical study of the reactions between vanadium−silicon heteronuclear oxide cluster anions with n-butane. *J. Phys. Chem. C* **114**, 12271-12279 (2010).

160. Ma, J.-B., Wu, X.-N., Zhao, X.-X., Ding, X.-L. & He, S.-G. Methane activation by $V_3PO_{10}^{\cdot+}$ and $V_4O_{10}^{\cdot+}$ clusters: a comparative study. *Phys. Chem. Chem. Phys.* **12**, 12223-12228 (2010).
